# Full-Length Transcriptome Survey and Expression Analysis of *Cassia obtusifolia* to Discover Putative Genes Related to Aurantio-Obtusin Biosynthesis, Seed Formation and Development, and Stress Response

**DOI:** 10.3390/ijms19092476

**Published:** 2018-08-21

**Authors:** Yin Deng, Hui Zheng, Zicheng Yan, Dongying Liao, Chaolin Li, Jiayu Zhou, Hai Liao

**Affiliations:** School of Life Science and Engineering, Southwest Jiaotong University, Chengdu 610031, China; DY1099438511@163.com (Y.D.); zh17828115685@163.com (H.Z.); yanzichh@163.com (Z.Y.); ldy20134237@163.com (D.L.); konekonelcl@163.com (C.L.)

**Keywords:** aurantio-obtusin biosynthesis, *Cassia obtusifolia*, organ, qRT-PCR, seed-specific genes, SMRT sequencing

## Abstract

The seed is the pharmaceutical and breeding organ of *Cassia obtusifolia*, a well-known medical herb containing aurantio-obtusin (a kind of anthraquinone), food, and landscape. In order to understand the molecular mechanism of the biosynthesis of aurantio-obtusin, seed formation and development, and stress response of *C. obtusifolia*, it is necessary to understand the genomics information. Although previous seed transcriptome of *C. obtusifolia* has been carried out by short-read next-generation sequencing (NGS) technology, the vast majority of the resulting unigenes did not represent full-length cDNA sequences and supply enough gene expression profile information of the various organs or tissues. In this study, fifteen cDNA libraries, which were constructed from the seed, root, stem, leaf, and flower (three repetitions with each organ) of *C. obtusifolia*, were sequenced using hybrid approach combining single-molecule real-time (SMRT) and NGS platform. More than 4,315,774 long reads with 9.66 Gb sequencing data and 361,427,021 short reads with 108.13 Gb sequencing data were generated by SMRT and NGS platform, respectively. 67,222 consensus isoforms were clustered from the reads and 81.73% (61,016) of which were longer than 1000 bp. Furthermore, the 67,222 consensus isoforms represented 58,106 nonredundant transcripts, 98.25% (57,092) of which were annotated and 25,573 of which were assigned to specific metabolic pathways by KEGG. *CoDXS* and *CoDXR* genes were directly used for functional characterization to validate the accuracy of sequences obtained from transcriptome. A total of 658 seed-specific transcripts indicated their special roles in physiological processes in seed. Analysis of transcripts which were involved in the early stage of anthraquinone biosynthesis suggested that the aurantio-obtusin in *C. obtusifolia* was mainly generated from isochorismate and Mevalonate/methylerythritol phosphate (MVA/MEP) pathway, and three reactions catalyzed by Menaquinone-specific isochorismate synthase (ICS), 1-deoxy-d-xylulose-5-phosphate synthase (DXS) and isopentenyl diphosphate (IPPS) might be the limited steps. Several seed-specific CYPs, SAM-dependent methyltransferase, and UDP-glycosyltransferase (UDPG) supplied promising candidate genes in the late stage of anthraquinone biosynthesis. In addition, four seed-specific transcriptional factors including three MYB Transcription Factor (MYB) and one MADS-box Transcription Factor (MADS) transcriptional factors) and alternative splicing might be involved with seed formation and development. Meanwhile, most members of Hsp20 genes showed high expression level in seed and flower; seven of which might have chaperon activities under various abiotic stresses. Finally, the expressional patterns of genes with particular interests showed similar trends in both transcriptome assay and qRT-PCR. In conclusion, this is the first full-length transcriptome sequencing reported in Caesalpiniaceae family, and thus providing a more complete insight into aurantio-obtusin biosynthesis, seed formation and development, and stress response as well in *C. obtusifolia*.

## 1. Introduction

The seeds of many pharmaceutical plants, such as *Prunus armeniaca* [1], *Ziziphi spinosae* [2], *Lycium barbarum* [3], and *Lepidium apetalum* [4], have been widely used as traditional medicines for centuries. Many bioactive metabolites, such as alkaloid, terpenoid, flavonoid, and anthraquinone, have been reported in the seeds of plants [5,6]. Furthermore, the seeds are breeding organs in establishment and persistence of medical plants, such as *Panax ginseng*, *Forsythia suspensa*, and *Alisma plantago-aquatica* [7]. Drought, high salinity, and extreme temperature are the major limiting factors of seed formation, development, and germination [8]. Therefore, the genetic background of the seed would underlay foundation for exploring the biosynthesis of bioactive metabolites in the seed, the molecular mechanism of seed formation and development, and stress response of the seed as well.

*Cassia obtusifolia* L., a leguminous annual plant, is easily grown, widely cultivated in subtropical and tropical countries in Asia and Africa. The seed of *C. obtusifolia*, called as Jue-ming-zi, was introduced in “Shen nong ben cao jing”, the earliest book on Chinese herbal medicine in the world, for the first time in the early 17th century. It was used as medicine and food materials in many traditional Chinese medical (TCM) prescriptions. Meanwhile, it is a healthy tea in China and Korea. In addition, it is used as food for animals and human to supplement the inadequate protein intake in India [9]. For medical purpose, it has been used to treat dizziness and headache, as well as benefit the eyes by anchoring and nourishing the liver. Recently, it has been reported to have neuroprotective effects in brain disease models [10]. The water extract of seed also enhanced antioxidant activity in hyperlipidemic rats induced by a high-fat diet [11]. Moreover, the seed and its constituents had potential antidiabetic activity and the possible mechanism was through increasing the insulin-provoked glucose uptake and inhibiting the protein tyrosine phosphatases 1B and *á*-glucosidase activities in human HepG2 cells [12]. The seed of *C. obtusifolia* contained a variety of bioactive anthraquinones, which were mainly responsible for the pharmacological action ascribed to them [7,13,14,15]. It was reported by the Chinese Pharmacopeia in 2015 that aurantio-obtusin, a kind of anthraquinone, was the most significant active gradient and regarded as the quality marker to evaluate the pharmaceutical value of *C. obtusifolia*. As aurantio-obtusin was a specific component found in *Cassia* plants, *C. obtusifolia* might be considered as a kind of model plant to research the biosynthesis of anthraquinone. The phytochemical analysis of *C. obtusifolia* extracts showed that aurantio-obtusin distributed mainly in the seed, while only a few contents of aurantio-obtusin in the root, leaf, flower, and stem. Therefore, it was a reasonable protocol to screen genes that catalyzed the limiting steps involving with the biosynthesis of aurantio-obtusin by comparing the expression difference of candidate genes between seed and other organs.

In addition, the seed of *C. obtusifolia* was harvested and used as the breeding organ. Therefore, it is important to study the seed formation and development of *C. obtusifolia*. Previous genetic analysis indicated that the seed formation and development of plants was controlled by several transcription factors, which were possibly involved in the abiotic stimuli, secondary and hormone metabolisms, and development [16]. Finally, *C. obtusifolia* is cultivated as a kind of landscape plant due to its ability to survive in a variety of environments, and thus its response to various abotic stresses become a noticeable issue. In higher plants, numerous genes, such as the Hsp20s family, have been reported to respond to these abiotic stresses [17]. These results provided a basis for further analysis of the molecular mechanisms of seed formation and development, and stress response.

To date, the molecular mechanism of biosynthesis of aurantio-obtusin, seed formation and development, and stress response in *C. obtusifolia* remained poorly understood due to the shortage of genomic information. Only seed transcriptome sequence information, based on short-read next-generation sequencing (NGS) technology, has been obtained from *C. obtusifolia* by our lab [18]. However, the fundamental limitation for NGS was the short sequencing products, which required assembly and led to a very small proportion of the assembled transcripts, and misassembly as well. Furthermore, short reads from NGS made it very difficult for transcripts containing repetitive elements and high GC contents. Therefore, these efforts cannot provide more help to obtain the satisfied genetic background for further research. In recent years, single-molecule real-time (SMRT) sequencing technology, referring to the use of third generation sequencing platforms to sequence cDNA, provided researchers with an efficient way to obtain the full-length cDNA sequences and had been used in non-model plants. Here, the NGS and SMRT sequencing technology were applied to characterize the transcriptome from seed, leaf, flower, stem, and root of *C. obtusifolia*. The transcriptome data would serve as a genetic platform for the researches of *C. obtusifolia*. Furthermore, the identified transcripts provided candidate target genes to discover the biosynthesis of aurantio-obtusin, the molecular mechanism of seed formation and development, and stress response in *C. obtusifolia*.

## 2. Results

### 2.1. Combined Sequencing Approach to the Various Organs of C. obtusifolia

To identify and differentiate the seed transcriptome from that of the rest organs, NGS and SMRT sequencing platforms were undertaken simultaneously. First, 15 mRNA samples from five different organs (seed, root, stem, leaf, and flower, each in triplicate) were subjected to the Illumina 4000 platform, with 361,427,021 clean reads (108.13 Gb) produced. Second, full-length cDNAs from five pooled poly (A) RNA samples were normalized and subjected to an SMRT sequencing using the PacBIO RS II platform. In total, 601,168 polymerase reads were generated. After filtering the adapter sequence from polymerase reads and removing sequences shorter than 50 bp, 4,315,774 subreads with sequence representing 9.66 Gb bases were obtained. Next, 282,022 ROIs were successfully extracted with mean length of 2458 bp, quality of 0.92, and 10 passes. All ROIs were further classified into 136,567 full-length nonchimeric and 114,185 non-full-length sequences with differential patterns of length distribution (Figure S2). Based on the clustering algorithm of IEC, we got 67,222 consensus isoforms with a mean length of 2250 bp, mainly generated from the 1–2 kb, 2–3 kb, and 3–6 kb libraries, as it proved to be too difficult to produce consensus isoforms from the <1 kb and >6 kb libraries, because of their shorter and larger insert lengths. The 67,222 consensus isoforms were composed of 54,533 high-quality transcripts and 12,689 low-quality transcripts which were further corrected using the Illumina reads to improve quality (Table 1). Then, after removing the redundant sequences for all high-quality transcripts and corrected low-quality transcripts using CD-HIT (c = 0.90), 58,106 nonredundant transcripts were produced. Besides those coding for proteins, LncRNAs (more than 200 bases) were predicted using CPC, CNCI, pfam, and CPAT analysis, 775 of predicted LncRNAs were able to match four databases. In total, 2712 alternative splicing events were detected from the nonredundant transcripts based on the de novo alignment method. 

Our previous NGS result indicated that the unigenes assembled from the Illumina short reads by Trinity largely did not represent full-length cDNAs [18].Approximately 78.36% of the assembled unigenes from NGS reads were <1000 bases, whereas only 9.23% of the consensus isoforms from the PacBIO reads were <1000 bases (Table 1). Indeed, the mean full-length read lengths from the different libraries (<1, 1–2, 2–3, 3–6, and >6 kb) produced by SMRT sequencing were 897, 1371, 2457, 3485, and 9064 bases, respectively (Table 1). Nevertheless, from this study, it seems that the use of NGS data to correct the low-quality SMRT reads may be a proper strategy. 

**Figure 1:**
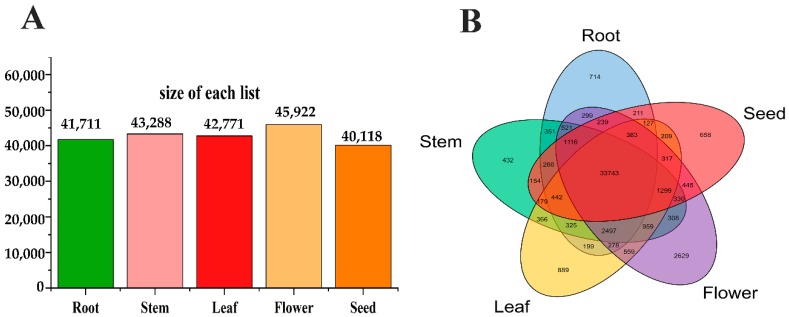
Specially expressed genes across five various organs of *C. obtusifolia*. (**A**) The gene profiles of five various organs. (**B**) Venn diagram for the organ-specific transcripts.

In total, from the NGS data, 40,118, 41,711, 43,288, 42,771, and 45,922 transcripts were found in seed, root, stem, leaf, and flower, respectively. Moreover, using a cut-off of FPKM > 10, expression from 5870 distinct genes was detected in the seed, with 7877 expressed in the root, 9023 in the stem, 8114 in the leaf, and 9082 in the flower. In addition, 658 genes were specially expressed in a single seed organ, 714 in the root, 432 in the stem, 889 in the leaf, and 2629 in the flower (Figure 1). Thus, it is possible to distinguish between the transcriptomes from each of these various organs. Both SMRT sequencing data and Illumina Hiseq 4000 data have been deposited in the Sequence Read Archive (SRA) of the National Center for Biotechnology Information (NCBI) under accession numbers SRP144670.

### 2.2. Functional Annotation Based on Searches against Public Databases

**Figure 2:**
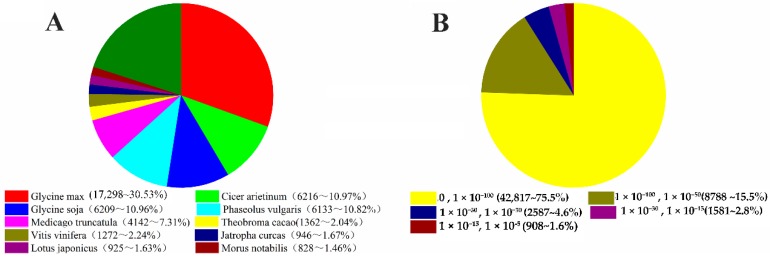
Characteristics of homology search of genes against the NR database. (**A**) Species distribution is shown as percentage of the total homologous gene hits. All of the top five species are Leguminosae. (**B**) E-value distribution of BLAST hits for each transcript with a cut-off of E-value of 1 × 10^−5^.

For annotation, distinct gene sequences were first searched using BLASTX against the Nr, Swiss-Prot, and KEGG databases. The domain/family searches were conducted against the COG, KOG, and Pfam database at NCBI using BLASTX. To functionally categorize the *C. obtusifiolia* transcripts based on the Nr annotation, gene ontology analysis was conducted. Using this approach, 57,092 nonredundant transcripts (98.25%) returned a significant BLAST result (Table 2). The annotation ratio of transcripts from SMRT platform was more than that (85%) from NGS platform [19], as more full-length sequences in the former platform supply better Blast results. The numbers of best BLASTX and domain hits for the unigene sequences in each of the databases are summarized in Table 2. A total of approximately 99.28% (56,681) of the *C. obtusifolia* transcripts were found to be homologs in the NR database with an e-value smaller than the cutoff. The annotated sequences had the greatest homology with those in *Glycine max* (17,298, 30.53%), followed by *Cicer arietinum* (6216, 10.97%), *Glycine soja* (6209, 10.96%), *Phaseolus vulgaris* (6133, 10.82%), *Medicago truncatula* (4142, 7.31%), *Theobroma cacao* (1362, 2.40%), *Vitis vinifera* (1272, 2.24%), *Jatropha curcas* (946, 1.67%), *Lotus japonicas* (925, 1.63%), and *Morus notabilis* (828, 1.46%). The remaining 20.00% sequences were matched to other plants. A comparative analysis of the e-value distribution of hit unigenes shows that 75.5% of *C. obtusifolia* transcripts have the highest homology with an e-value cutoff smaller than 1 × 10^−100^ (Figure 2).

### 2.3. Functional Analysis of CoDXS and CoDXR Genes

**Figure 3:**
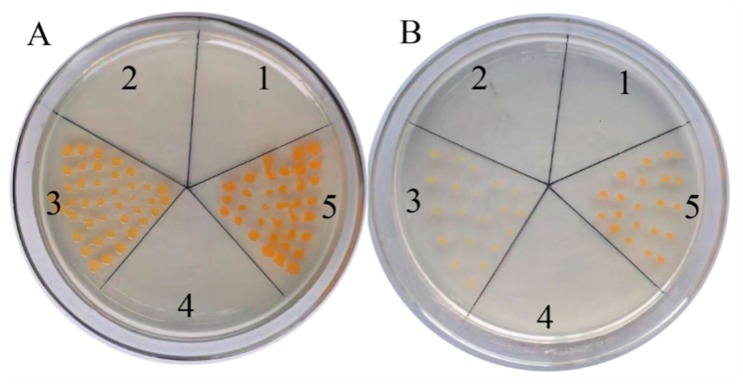
Function demonstrantion of *CoDXS* (**A**) and *CoDXR* (**B**) in *E. coli*. The *E. coli* Top 10 was transformed with pTrc-*Co*DXS and pAC-BETA harboring *CoDXS* (A5) or pTrc-CoDXR and pAC-BETA harboring *CoDXR* (B5) pTrc and pAC-BETA (A3, B3), pAC-BETA (A4, B4), pTrc-*Co*DXS (A2), pTrc-*Co*DXR (B2), and pTrc (A1, B1), respectively.

It was necessary to evaluate the accuracy of sequences from the *C. obtusifolia* transcriptome, two full-length genes annotated as *CoDXS* and *CoDXR*, and were then directly used for functional identification. We used a kind of genetically engineered *E. coli*, which provided a visible color assay to examine the biological function of genes/enzymes involved in MEP pathway, such as DXS, DXR, IPPS, PSY, etc., from various plants including *Amomum villosum* [20], *Vitis vinifera* [21], and *Tripterygium wilfordii* [22]. To confirm the function of *CoDXS*, the plasmids pTrc-*Co*DXS and pAC-BETA were cotransformed into *E. coli* Top10. The cotransformed *E. coli* cells containing pTrc-*Co*DXR and pAC-BETA showed more intense yellow color than the control containing the empty vector pTrc and pAC-BETA (Figure 3A). The results indicated that the *CoDXS* enzymes were functionally active and involved in carotenoid biosynthesis in recombinant *E. coli* cells. *E. coli* harboring the single vector pAC-BETA could not grow due to the lack of ampicillin resistance gene.

Also, the cotransformations were performed using the method mentioned above to demonstrate that the *CoDXR* encodes the anticipative functional protein. The result showed that *E. coli* containing pTrc-*Co*DXR and pAC-BETA accumulated significantly higher saffron yellow color than the control, which was similar to that of *CoDXR* (Figure 3B). These results indicated that the sequences obtained from the hybrid platform were accurate and could be used directly for genetic manipulation.

### 2.4. Gene Ontology (GO) Classification

**Figure 4:**
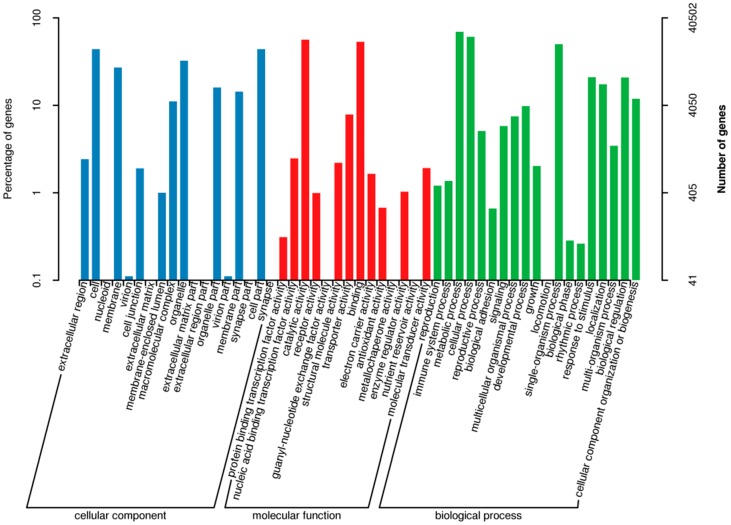
Gene ontology classification. Note: Gene ontology was summarized as three main categories: cellular component (left panel), molecular function (middle panel), and biological process (right panel). The percentage (left *Y*-axis) and number (right *Y*-axis) of genes were also showed.

Based on sequence homology, 40,502 transcripts can be assigned to GO classes to classify the functions of the predicted *C. obtusifolia* genes. The assigned GO terms were summarized into the three main GO categories, biological process, cellular component, and molecular function, and then into 50 functional categories (shown in Figure 4). Biological process comprised 116,967 (47.16%) GO annotations and was the largest cluster, followed by cellular component 78,824 (31.78%), and molecular function (52,256 and 21.07%). Under the molecular function category, catalytic activity (22,830 and 43.69%) represented the most abundant term. About 21,114 and 22,830 transctipts in “metabolic process” and “catalytic activity” category, respectively, suggesting this study may allow for the identification of novel genes involved in the secondary metabolite synthesis pathways. KEGG pathway mapping of *C. obtusifolia* transcriptome assembly resulted in transcripts assigned to KEGG pathway. 1214 transcripts involved in biosynthesis of secondary metabolites were found (shown in Table 3). Among them, 363, 79, and 165 transcripts were annotated from “phenylpropanoid biosynthesis”, “steroid biosynthesis”, and “terpenoid backbone biosynthesis” pathways, which provide precursors for the biosynthesis of aurantio-obtusin.

### 2.5. Localization of Aurantio-Obtusin Accumulation and Gene Profiles Involved in Aurantio-Obtusin Biosynthesis

**Figure 5:**
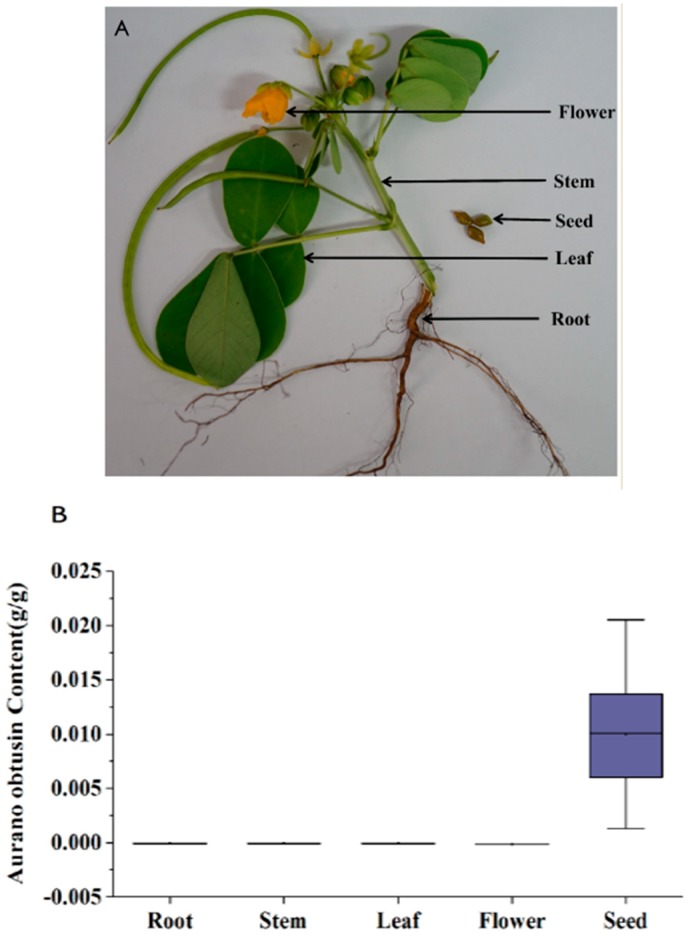

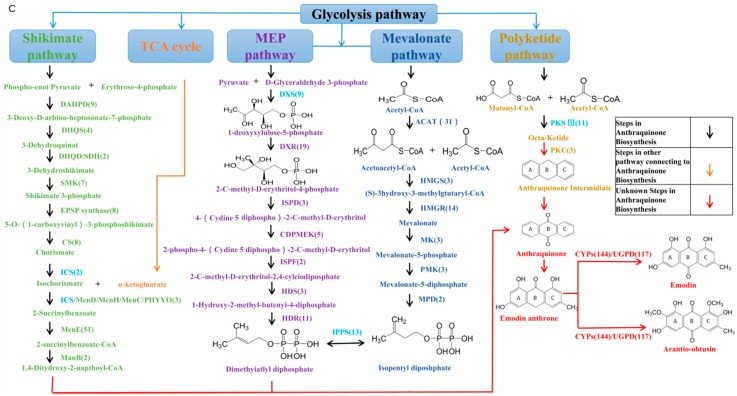
Various organs, aurantio-obtusin biosynthesis-related genes, and phytochemical analysis of *C. obtusifolia*. (**A**) Five various organs of *C. obtusifolia.* (**B**) Aurantio-obtusin levels in the five various organs. (**C**) Proposed biosynthetic pathway and transcripts involved in the biosynthesis of aurantio-obtusin in *C. obtusifolia*. Note: DXS, 1-deoxy-d-xylulose-5-phosphate synthase; DXR, 1-deoxy-d-xylulose 5-phosphate reductoisomerase; ISPD, 2-C-methyl-d-erythritol 4-phosphate cytidylyltransferase; CDPMEK, 4-diphosphocytidyl-2-C-methyl-d-erythritol kinase; ISPF, 2-C-methyl-d-erythritol 2,4-cyclodiphosphate synthase; HDS, (E)-4-hydroxy-3-methylbut-2-enyl diphosphate synthase; HDR, 4-hydroxy-3-methylbut-2-enyl diphosphate reductase; HMGS, 3-hydroxy-3-methylglutaryl CoA synthase; HMGR, Hydroxymethylglutaryl-CoA reductase; MK, Mevalonate kinase; PMK, Phosphomevalonate kinase; MPD, Mevalonate pyrophosphate decarboxylase; IPPS, Isopentenyldiphosphate isomerase; DAHPS, 3-deoxy-d-arabino-heptulosonate 7-phosphate synthase; DHQS, 3-dehydroquinate synthase; SDH, Shikimate 5-dehydrogenase; SMK, Shikimate kinase; MenF, isochorismate synthase; PKS III, polyketide synthase III; PKC, polyketide cyclase; MenD, 2-succinyl-5-enolpyruvyl-6-hydroxy-3-cyclohexene-1-carboxylate synthase; MenH, 2-succinyl-6-hydroxy-2,4-cyclohexadiene-1-carboxylate synthase;MenB, naphthoate synthase; MenC, *o*-succinylbenzoate synthase; MenE, o-succinylbenzoic acid-CoA ligase; CYPs included CYP450, CYP450 monooxygenase and NADPH-CYP450 reductase. DMT, di-O-methyltransferase; UDPG, UDP-glycosyltransferase. The transcripts numbers of each gene are represented in the brackets.

It is the seed of *C. obtusifolia* that is used in TCM, accounting for a model medicinal herb to study the aurantio-obtusin biosynthesis. Five various organs, including seed, root, leaf, flower, and stem of *C. obtusifolia*, were collected (Figure 5A). The HPLC results indicated that aurantio-obtusin was located in seed (1% of aurantio-obtusin content), whereas root, leaf, flower, and stem did not have aurantio-obtusin compound (Figure 4B). These results suggested that aurantio-obtusin biosynthesis is mainly carried out in this organ, providing a potential basis for colocation analysis.

The biosynthesis of aurantio-obtusin in plants is derived from studies on anthraquinone biosynthesis in other species. Biosynthesis of anthraquinones was studied in the Rubiaceae family such as *Lithospermum*, *Morinda*, and *Rubia* species [23,24,25]. One of the remarkable features of anthraquinone biosynthesis in higher plants is that they are derived from a variety of different pathways and hence difficult to elucidate. Anthraquinones in plants are derived biosynthetically by a combination of the isochorismate and mevalonic acid (MVA)/2-methyl-d-erythritol 4-phosphate (MEP) pathways [26]. Anthraquinone is made up of three benzene rings named by A, B, and C. In the early stage of anthraquinones formation, the rings of A and B are derived from 1,4-dihydroxy-2-naphthoc acid via isochorismic acid and α-ketoglutaric acid, whereas ring C is derived from isopentenyl diphosphate (IPP)/3,3-dimethylallyl diphosphate (DMAPP) via the MVA/MEP pathway. To date, though a variety of genes involving Isochorismate, MVA, and MEP pathways have been identified in plants, a limited number of genes encoding enzymes in the biosynthesis of anthraquinone have been identified from anthraquinone-containing plants, including DXS encoding 1-deoxy-d-xylulose 5-phosphate synthase from *Morinda citrifolia* [27], DXR encoding, ICS encoding isochorismate synthase from *Rubia tinctorum* [28] and *Rubia cordifolia* [28], OSBS encoding *o*-succinylbenzoate synthase from *Rubia cordifolia* [29], SK encoding shikimate kinase from *Cassia obtusifolia* [30], OSBL encoding *o*-succinylbenzoate ligase from *Rubia cordifolia* [30], IPPI encoding isopentenyl-diphosphate isomerase in *Cinchona robusta* [31] and *Rubia cordifolia* [29], and Polyketide synthases III from *Rheum emodi* [32] and *Aloe arborescens* [33]. In the late stage of anthraquinones biosynthesis, the backbone of anthraquinones then undergoes various modifications mediated by cytochrome P450s, SAM-dependent methyltransferases, UDP-glycosyltransferases (UDPG), and other enzymes. To date, no genes involved in the late steps of anthraquinones biosynthesis have been identified from *C. obtusifolia* or other anthraquinone-producing plants.

The biosynthesis of anthraquinone shares isochorismate pathway with phenylpropanoid and MVA/MEP pathway with sterol and terpenoids. Isochorismate pathway leads to produce isochorismate which forms a substrate for 1,4-dihydroxy-2-napthoyl-CoA, a precursor for anthraquinone backbone. We have identified 33, 34, 34, 33, and 33 transcripts in seed, root, stem, leaf, and flower libraries, respectively, for six enzymes involved in the Isochorismate pathway. Similarly, for four enzymes in the menaquinone pathway, we have identified 42, 45, 45, 44, and 45 transcripts in seed, root, stem, leaf, and flower libraries, respectively. The MVA and MEP pathways produced dimethylallyl diphosphate, another precursor of the anthraquinone backbone. In our study, there were 40, 40, 40, 43, and 47 transcripts in seed, root, stem, leaf, and flower libraries, respectively, for six enzymes involved in the MVA pathway. In the MEP pathway, there were 46, 48, 51, 56, and 54 transcripts in seed, root, stem, leaf, and flower libraries, respectively. Biosynthesis of anthraquinone is also known to be from acetyl-CoA and malonyl-CoA through polyketide pathway in plants. Polyketide synthase III might be a key enzyme involved in the polyketide pathway [34]. In our study, there were 12, 12, 13, 13, and 13 transcripts in seed, root, stem, leaf, and flower libraries, respectively, encoding the enzymes involved in the polyketide pathway. In the late stage of anthraquinone biosynthesis, CYP450s, SAM-dependent methyltransferases and UDPG are mainly responsible for the modification of anthraquinone backbone to produce a variety of anthraquinone compounds [35]. CYP450s are member bound hemoproteins involved in biosynthesis of secondary metabolites such as phenylpropanoid, flavonoid, steroid with sterol, and terpenoids. CYP450s catalyze oxidative reactions, including hydroxylations, epoxidation, dealkylation, dehydration, and carbon—carbon bond cleavage of metabolites. According to the BLAST annotation, 135, 135, 151, 144, and 152 CYP450s were found in seed, root, stem, leaf, and flower libraries encoding for CYP450, CYP450 monooxygenase, and NADPH-CYP450 reductase enzymes, respectively. SAM-dependent methyltransferases make use of the methyl group from SAM and catalyzes the methylation of the hydroxyl group of substrate to produce methyl secondary metabolites, such as phenylpropanoids [36]. Seventy-seven, 83, 80, 76, and 91 SAM-dependent methyltransferases were discovered in seed, root, stem, leaf, and flower libraries, respectively. In general, glycosylation takes place at the end of secondary metabolites biosynthesis and results in increased stability, water solubility, and sometimes biological activity. In nature, UDPG catalyzes glycosylation at the site of hydroxyl group. In our study, there were 82, 90, 96, 96, and 99 UDPGs in seed, root, stem, leaf, and flower libraries, respectively. These CYP450s, SAM-dependent methyltransferases and UDPGs genes are therefore promising candidates for anthraquinone biosynthesis, and further enzyme assays are required to identify the function of these candidate genes (Figure 5C, Table S2).

### 2.6. Expression Analysis Indicates Colocalization of Aurantio-Obtusin Biosynthesis and Accumulation

**Figure 6:**
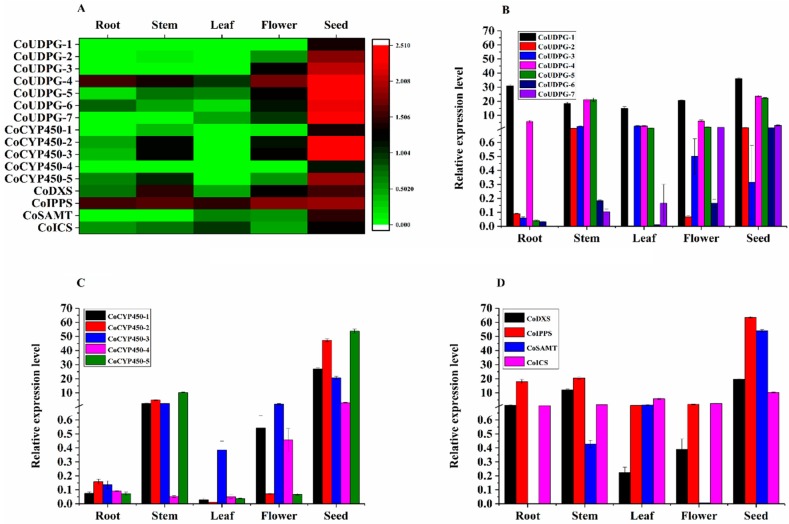
Heat map and following qRT-PCR analysis of the various seed-specific CYPs, SAM-methyltransferases, UPDGs, as well as ICS, DXS, and IPPS with the highest expression level in the seed and different organs, respectively. ICS, DXS, and IPPS were known to be the potential key enzymes involved in the anthraquinone biosynthesis. (**A**) Heat map depicting differential gene expression of seed. (**B**–**D**) qRT-PCR analysis.

Given the clear accumulation of aurantio-obtusin in seed, we hypothesize that aurantio-obtusin biosynthesis occurs mainly in this organ. Accordingly, the remainder of the genes encoding enzymes involved in the aurantio-obtusin biosynthesis might be expected to exhibit a similar (relative) high expression level in seed. Consistent with the localized production of aurantio-obtusin in the seed, analysis of expression levels revealed that 20 transcripts involved in isochorismate pathway were expressed in the seed with FPKM > 10. Of which, at least one isoform of each of the enzymes (DAHPS, DHQS, SDH, SMK, EPSP, CS, ICS, MenE, and MenB) showed somewhat higher (relatively high) expression in the seed than other organs, whereas DHQS, SDH, EPSP, and ICS showed highest expression level in the seed. In MVA pathway, seven transcripts were expressed in the seed with FPKM > 10. Of which, HMGR also showed somewhat higher expression in seed than other organs, whereas one isoform of ACAT and MPD showed the highest expression level in seed. In MEP pathway, 15 transcripts encoding DXS, DXR, CDPMEK, ISPF, HDR, and IPPS were expressed in the seed with FPKM > 10, of which one isoform of DXS and IPPS, respectively, showed the highest expression level in seed. In polypeptide pathway, three transcripts encoding PKS III and PKC showed high expression with FPKM > 10, but not the highest expression level in the seed. It was believed that there are three limiting steps, which are catalyzed by Isopentenyldiphosphate isomerase (IPPS), 1-deoxy-Dxylulose-5-phosphate synthase (DXS), and isochorismate synthase (ICS), respectively, in the early stage of anthraquinones formation (14–16). Therefore, the observed highest expression levels of ICS, DXS, and IPPS in seed is consistent with those role in anthraquinone production and was verified by followed qRT-PCR that showed similar expression pattern with that in the transcriptome (Figure 6). Moreover, based on the differential expression analysis, ACAT, DXS, EPSP, and ICS were found to be upregulated in the seed (Table S4). As a result, comparing with IPPS, ICS, and DXS might be regarded as the important regulatory target for the aurantio-obtusin biosynthesis in *C. obtusifolia*. In addition, both EPSP and ACAT might be the candidate key enzymes involved in the aurantio-obtusin biosynthesis; the hypothesis still needs verification by further transgenic research.

CYP450s, SAM-dependent methyltransferases and UDPG are mainly involved in the late stage of anthraquinone biosynthesis. Consistent with the expanded nature of the CYP superfamily in plants, a total of 135 CYPs were identified from the seed of *C. obtusifolia*. Among them, 20 were expressed in the seed with an FPKM > 10, with nine exhibiting upregulated expression levels (and five quite specifically expressed) in seed. In present study, 17 SAM-dependent methyltransferases were found with FPKM > 10, with five members exhibited the highest expression level, in which F01.PB47348 showed specific expression in seed and was annotated as di-*O*-methyltransferase like protein. Di-*O*-methyltransferase catalyzed two sequential methylation reactions to form methyl metabolites. Aurantio-obtusin biosynthesis involved two methylation reactions after the formation of emodin, which is suggested to be the precursor of aurantio-obtusin. Therefore, F01.PB47348 was a strong candidate for a possible role in aurantio-obtusin biosynthesis; further functional characterization is necessary. 25 UDPGs were found to be highly expressed in seed with FPKM > 10, with 16 exhibiting the highest expression level (10 upregulated and seven quite specifically expressed) in the seed. Those transcripts that were related to anthraquinone biosynthesis and had high expression levels (with FPKM > 10 in seed) were summarized in Tables S3 and S4. The followed qRT-PCR results of these seed-specific CYPs, SAM-methyltransferases and UDPGs showed similar expression pattern with those in the transcriptome (Figure 6), except *Co*UDPG-1, which showed different expression pattern in root, stem, leaf, and flower, in both the qRT-PCR and transcriptome assay.

**Figure 7:**
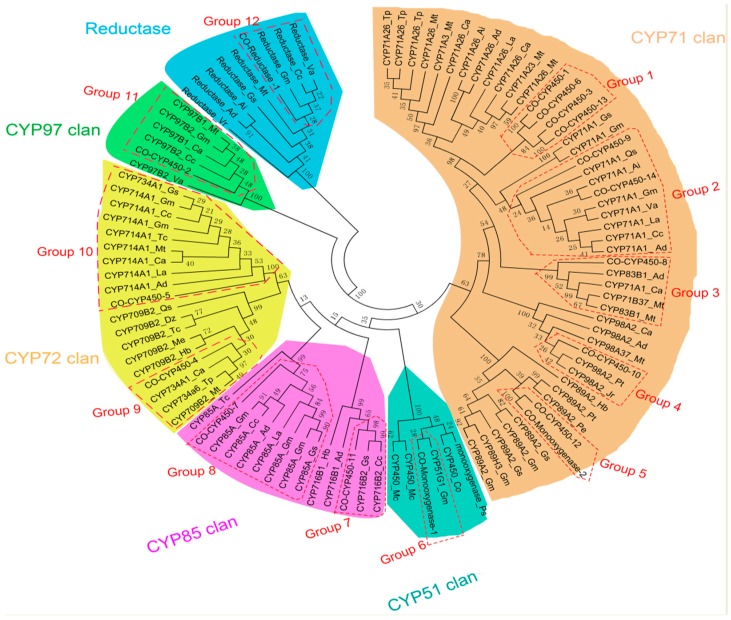
Phylogenetic analysis of seed-specific candidate transcripts coding CYPs associated with the late stage of anthraquinone biosynthesis with cytochrome P450s across different plant species. The nucleotide sequences of seed-specific candidate transcripts were translated into protein sequences, and subjected to multiple sequence alignment with 85 selected plant CYPs from different subfamilies (available online: http://drnelson.uthsc.edu/CytochromeP450.html).

Moreover, in order to predict possible function of these 20 CYPs, including 17 CYP450s, two CYP450 monooxygenases, and one CYP450 reductase, phylogenetic analysis was performed together with 85 representative CYP450s from different plant species (Figure 7, Table S5). To obtain an accurate functional prediction of CYPs, only those CYPs with sequence length over 600 bp were selected for phylogenetic analysis. As a result, 17 CYPs (the amino acid of the CYPs were listed in the supplementary data), except 35 (F01.PB1877, 3544, and 15474), were applied with phylogenetic analysis which showed nine out of the CYPs and one CYP450 monooxygenase being grouped in the CYP71-clan. Enzymes from the CYP71-clan are believed to undergo successive gene duplication events resulting in species-specific enzyme-clade, which, in turn promotes the new specialized metabolite biosynthesis in the plant. Within the CYP71-clan, 10 transcripts were clustered in four groups. In group 1, four transcripts (*Co*CYP450-1, 3, 6, and 13) showed high phylogenetic relationship with CYP71A26. The function of CYP71A26 might be involved in the ginsenosides and flavor compounds biosynthesis by catalyzing the C10 hydroxylation of α-pinene to myrtenol, and also in the response to heavy metal and salt stresses [37,38,39]. Interestingly, *Co*CYP450-1 was found to be a potential target gene of LncRNA (F01. PB55451), suggesting F01. PB55451 might play key role in the biosynthesis of secondary metabolites by regulating the mRNA content of *Co*CYP450-1. In group 2, two putative CYP450s (*Co*CYP450-9 and 14) were phylogenetically close to CYP71A1. It was demonstrated that the CYP71A1 from *Nepeta racemosa* and *Persea americana* participated in the monoterpenoids biosynthesis and was capable of oxidizing nerol and geraniol to the corresponding 2,3- and 6,7-epoxides [40]. In group 3, *Co*CYP450-8 showed close phylogenetic relationship with CYP83B1 that has been shown to catalyze the initial step of indole glucosinolate biosynthesis [41]. Group 4 included *Co*CYP450-10, which was phylogenetically related to CYP98A2. CYP98A2 belonged to the CYP98A family that catalyzed the meta-hydroxylation of p-coumarate derivatives, an important step in the phenylpropanoid pathway [42]. The last group in CYP71 clan, group 5 included *Co*CYP12 and *Co*CYP450 Monooxygenase-2, showing close phylogenetic relationship with CYP89A2. CYP89A2, a potential target enzyme of WAX INDUCER1 (HvWIN1) transcription factor, was reported to be involved in the pathogen and herbicide resistance by regulating the biosynthesis of the long-chain fatty acid biosynthesis [43,44]. There were two transcripts (*Co*CYP450-4 and 5) in the CYP72 clan. *Co*CYP450-4 was close to CYP734A6 and was a crucial brassinosteroid (BR) catabolic gene regulated by a KNOX transcription factor [45]. *Co*CYP450-5 was close to CYP714A1, a member of CYP72 clan, which has been reported to be involved in the biosynthesis of diterpenoid phytohormone GA by conversing GA12 to 12α-hydroxy GA12 [46]. In the CYP85 clan, *Co*CYP450-7 and 11 were close to CYP85A and CYP716B2, respectively. CYP85A1 catalyzed several essential reactions necessary for the production of castasterone and was involved in the oxidation steps necessary for the biosynthesis of BR [47]. CYP716B2 was a member of CYP716B family which was functionally characterized as taxoid 9*á* hydroxylase in *Gingko biloba* [48]. There was only one member in CYP51 and CYP97 clan, respectively. In the CYP51 clan, *Co*CYP450 Monooxygenase-1 showed a close relationship with CYP51G1. CYP51G1, also named as sterol 14alpha-demethylase, catalyzed the first step following cyclization in sterol biosynthesis, leading to the formation of precursors of steroid hormones, including brassinosteroids, in plants [49]. *Co*CYP450-2 showed a close relationship with CYP97B2, encoding enzymes downstream of â-carotene biosynthesis [50]. *Co*CYP450 reductase 1 was homologous with cytochrome P450 reductases that provides electrons to CYPs and takes part in the biosynthesis of a huge number of different metabolites, such as sterol and monoterpenoid [51].

### 2.7. Expression Pattern of Transcription Factors (TFs) in Seed

There were 303 TFs with FPKM > 10 in seed, mainly including 101 Zinc finger, 24 MYB, 24 ARF, 19 NAC, 18 Homeobox, 18 bHLH, and 15 NF TFs (Table S3). Zinc finger TFs possessed the zinc finger motif and played an extensive role in seed germination, secondary wall formation, and response to heat stress. MYB TFs were involved in controlling various processes like responses to biotic and abiotic stresses, development, differentiation, secondary metabolism, and defense [50,52,53]. In plants, the Auxin response factor (ARF) transcription factor family regulates gene expression in response to auxin and plays essential part in the root and gametophyte [54,55]. NAC TFs regulated a wide variety of processes in seed size, secondary wall biosynthesis, and secondary metabolites biosynthesis [56,57,58]. Homeobox TFs were important regulators of meristem function and were regulated tightly in a complex network of TFs [59]. WRKY TFs had diverse biological functions in seed and trichome development, embryogenesis, as well as additional developmental and hormone-controlled processes [60]. bHLH TFs were widely distributed in eukaryotic organisms and were thought to be one of the largest families of regulatory proteins, which played crucial roles in plant development [61]. NF TFs had been implicated in endosperm development, establishment of the legume-rhizobia symbiosis, and responding to abiotic stresses [62,63,64]. Meanwhile, there might be a role for alternative splicing in regulating seed formation and development more generally (Figure S3). First, nine ZF TFs exhibited alternative splicing, with only one isoform expressed with FPKM > 10 in seed. In addition, one SBP and one bHLH TFs were observed with only one isoform with FPKM > 10 in seed, suggesting that the regulation of alternative splicing may play a role in controlling the seed formation and development processes (Table S3).

**Figure 8:**
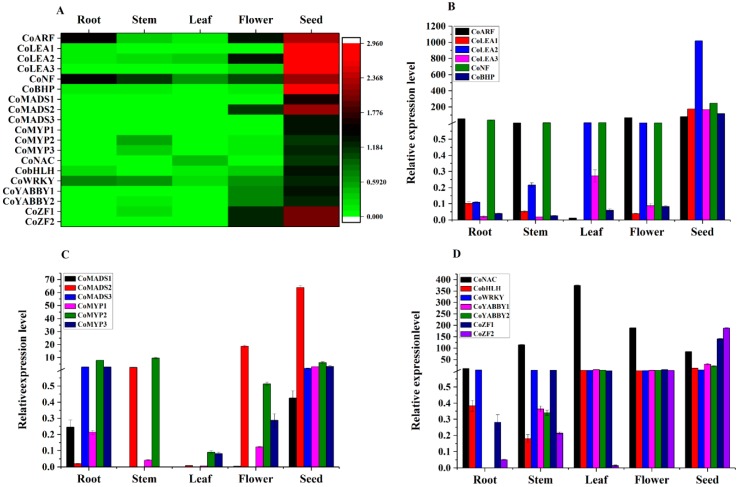
Transcriptome and following qRT-PCR analysis of the TFs in different organs including seed, root, stem, leaf and flower, respectively. (**A**) Heat map depicting of seed-specific TFs. (**B**–**D**) qRT-PCR analysis of seed-specific TFs.

Of note, 58 TFs showed the highest expression level in the seed, mainly including 18 Zinc finger, 6 MADS, 5 MYB, 4 GATA, 4 NF, 3 ARF, 3 NAC, 3 LEA, and 3 Trihelix TFs (Table S3). Among them, 19 TFs were specifically expressed in seed, including 3 MYB, 3 MADS, 3 LEA, 2 YABBY, 2 Zinc finger, 1 ARF, 1 NF, 1 HBP, 1 NAC, 1 bHLH, and 1 WRKY TFs. Besides the TFs described above, MADS played crucial role in nearly all aspects of plant development and contributed to the increasing complexity of plants [65]. Late embryogenesis abundant (LEA) protein and Zinc finger TFs regulate seed germination together [66]. YABBY-like and LOL TFs regulate fruit size together [67]. GATA TFs, which are responsive to various environmental change, regulated seed and leaf development and differentiation [68,69,70]. Trihelix TFs were plant-specific transcription factors, which are known to have a wide range of functions in growth and development processes involving the flower, stomata, embryo, and seed, as well as abiotic and biotic stresses [71,72]. HB29 TFs, which improved the transcriptional activities of MED25 and NAC TFs, could increase drought tolerance by upregulating several stress-inducible genes [73]. A heatmap of 19 seed-specific TF genes, represented by FPKM values in different organs, was established by R-software and validated by further qRT-PCR. As a result, 18 seed-specific TFs, except the NAC TF, showed similar expression pattern with those in the transcriptome (Figure 8).

**Figure 9:**
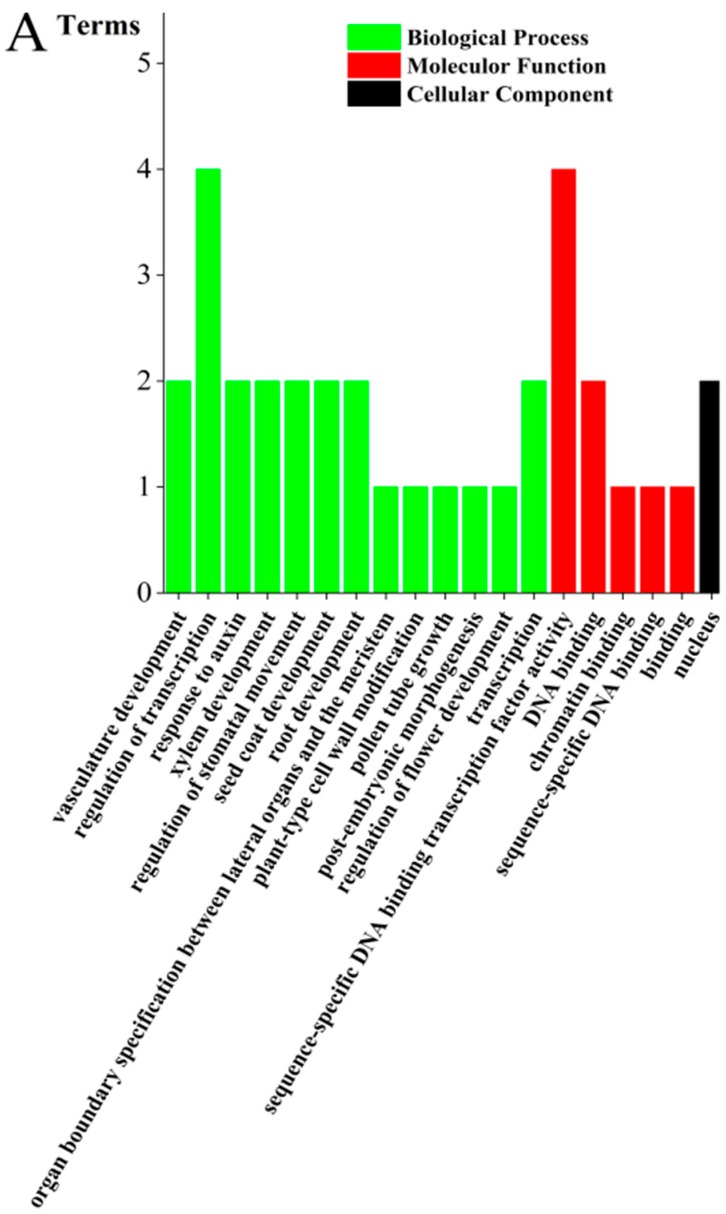

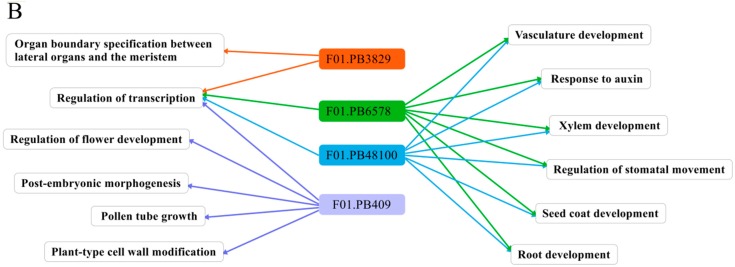
Gene ontology classification of seed-specific TFs. (**A**) The results were summarized in three main categories: cellular component, molecular function, and biological process. (**B**) The biological process of four TFs related to seed formation and development. F01.PB3829, F01.PB48100, F01.PB6578, and F01.PB409 represented CoMYB1, CoMYB2, CoMYB3, and CoMADS2, respectively.

Furthermore, those 18 verified seed-specific TFs were assigned to GO analysis to classify the predicted functions and six TFs were annotated and assigned at least one term in molecular function, cellular component, and biological process categories. The six TFs were further classified into 19 functional terms, providing an overview of ontology content (Figure 9A). In the biological process category regulation of transcription (GO:0006355) was the most highly represented term, while sequence-specific DNA binding transcription factor activity (GO:0003700) dominated the molecular function category, indicating those six seed-specific TFs which possessed extensive transcriptional regulatory activities. Among them, *Co*MYB1 (F01.PB3829), *Co*MYB2 (F01.PB48100), *Co*MYB3 (F01.PB6578), and *Co*MADS2 (F01.PB409), were suggested to be the strong candidate TFs involved in seed formation and development. F01.PB3829 might participate in organ boundary specification between lateral organs and the meristem (GO:0010199). F01.PB48100 and F01.PB6578 might take part in the vasculature (GO:0001944), xylem (GO:0010089), seed coat development (GO:0010214), and regulation of stomatal movement (GO:0010119) processes together. F01.PB409 was suggested to regulate plant-type cell wall modification (GO:0009827) and post-embryonic morphogenesis (GO:0009886) process (Figure 9B).

### 2.8. Expression Pattern of CoHsp20 Genes in Various Organs and under Different Stress

**Figure 10:**
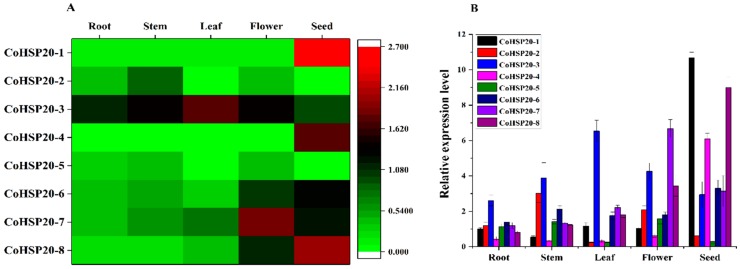
Heat map and following qRT-PCR analysis of the Hsp20 genes in different organs. (**A**) Heat map depicting differential gene expression levels. (**B**) qRT-PCR analysis.

It was regarded that abiotic and biotic stress would affect seed development and germination [17]. It was shown that small heat shock proteins (HSP20s) were a group of stress-responsive proteins mainly expressed in the seed and flower [74]. The HSP20s were ATP-dependent small chaperons with molecular weight ranging from 15 kDa to 42 kDa [75]. Hsp20s could avert protein denaturation, and thus maintain cellular homeostasis during abiotic stresses [76]. Hsp20s belonged to the heat shock protein family, including not only Hsp20s, but HSP100s, HSP90s, HSP70s, and HSP60s. Unlike other HSPs, the HSP20s exhibited extensive sequence variability, but a highly conserved 80–100 long amino acid sequence termed as á-crystalline C-terminal domains (ACD) [77]. The ACD contained two conserved regions, one in the N-terminal consensus region and the other is connected through a hydrophobic *â*6-loop at the C-terminal common region [78]. Hsp20s played an important role in plant stress response, such as heat, drought, salt, and cold. In this study, eight putative Hsp20 (*Co*Hsp20) genes were identified from the transcriptome of *C. obtusifolia*. A heatmap of eight *Co*Hsp20 genes, represented by FPKM values in different organs, was established by R-software (Figure 10A). Most of *Co*Hsp20s were expressed in one organ at least, except *Co*Hsp20*-5*, which was barely expressed in any organ. Some *Co*Hsp20 genes showed similar expression patterns in various organs. *Co*Hsp20*-1*, *4*, and *8* were specifically expressed in the seed. *Co*Hsp20*-6* and *7* showed relatively high expression high levels in seed and flower. *Co*Hsp20*-3* showed high expression level in most of organs, but relatively low expression level in the seed. *Co*Hsp20*-2* showed the highest expression level in the stem, but a lower expression level in seed and leaf. Among the eight *Co*Hsp20 genes six (except *Co*Hsp20*-2* and *5*) showed higher expression level in the seed and flower, suggesting their protective roles in the stress-sensitive reproductive organs. In order to demonstrate the reliability of the *C*oHsp20 genes expression profiles in the *C. obtusifolia* transcriptome, the qRT-PCR was used to investigate the relative expression level of eight *Co*Hsp20 genes in five various organs using *EF1á* as reference gene. As a result, expression trends were consistent for most of *Co*Hsp20s, except *Co*Hsp20*-6*, which showed different expression pattern in flower and stem, in both the qRT-PCR and transcriptome assay (Figure 10B).

**Figure 11:**
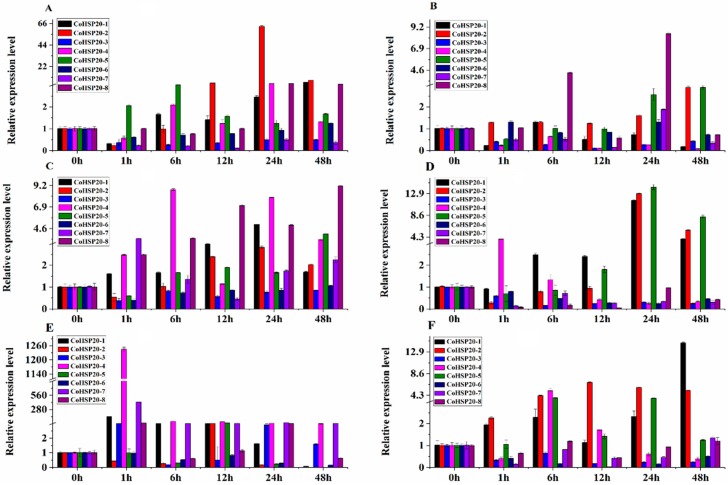
The relative expression level of various *Co*Hsp20 genes under heat (**A**), salt (**B**), drought (**C**), cold (**D**), ABA (**E**), and MeJA (**F**) treatment.

Meanwhile, in order to understand the metabolism of the eight Hsp20 genes under stresses, the sterile seedlings (30 days after germination) were treated with different stresses including heat, salt, drought, cold, ABA, and MeJA, and then used as samples to analyze the expression pattern of the eight Hsp20 genes by real-time RT-PCR. Generally, the relative expression level of the *Co*Hsp20 genes under all stresses fluctuated during 1 to 48 h treatments (Figure 11). Most of the *Co*Hsp20 genes were upregulated to heat stress, but *Co*Hsp20*-6* was downregulated after 24 h heat treatment. The relative expression levels of three *Co*Hsp20 genes (*Co*Hsp20*-1*, *4*, and *7*) were extremely upregulated (more than 100 fold) after 1 h heat treatment compared with control.

Although the Hsp20 family is generally induced by heat stress, we also investigated whether the family was involved in response to salt, drought, cold, ABA, and MeJA treatments. In general, the family was less sensitive under salt, drought, cold, ABA, and MeJA stresses than that under heat stress. Under salt stress, *Co*Hsp20*-2* was evidently regulated (63-fold) after 24 h treatment. *Co*Hsp20*-1*, *4*, *5*, and *8* reached the highest expression level after 48 h, 24 h, 6 h, and 24 h treatment, respectively, whereas *Co*Hsp20*-3* and *7* were downregulated after salt treatment. Only *Co*Hsp20*-2*, *5*, and *8* showed medium upregulated expression levels (2.89, 2.89, and 8.49 fold, respectively) under drought stress. *Co*Hsp20*-3*, *4*, and *7* were downregulated and *Co*Hsp20*-6* was not sensitive to drought stress. After cold treatment, *Co*Hsp20*-1*, *2*, *4*, and *5* were upregulated (14.66, 6.86, 5.18, and 3.77 fold, respectively) after 48 h, 24 h, 6 h, and 6 h, respectively, compared with control. *Co*Hsp20*-3* and *6* were downregulated and *Co*Hsp20*-8* was not sensitive to cold stress. Six genes (*Co*Hsp20*-1*, *2*, *4*, *5*, *7*, and *8*) showed medium upregulated expression levels (5.05, 2.82, 7.91, 4.02, 2.25, and 7.06, respectively) under ABA treatment after 24 h, 24 h, 24 h, 48 h, 48 h, and 12 h, respectively, but *Co*Hsp20*-3* and *6* showed no differences. *C*oHsp20*-1*, *2*, and *5* showed upregulated expression levels (11.55, 12.90, and 14.18 fold, respectively) after 24 h treatment with MeJA. *Co*Hsp20*-4* reached the highest expression level (3.88 fold) after 1 h treatment, whereas *Co*Hsp20*-3*, *6*, *7*, and *8* were downregulated under MeJA treatment. The differential expression patterns compared with those under heat stress indicated there were different response mechanisms of the *CoHsp20* family under various abiotic stress conditions.

## 3. Discussion

The long-history and widespread use of Juemingzi has led to intense interest in the biosynthesis of relevant bioactive compounds, seed formation and development, and to the molecular mechanism of the stress response as well. Aurantio-obtusin was the natural product of pharmaceuticalimportance and therefore regarded as the quality marker of *C. obtusifolia*. In the present study, seed-specific location of aurantio-obtusin was observed and therefore suggested that the seed was an important organ for the study of aurantio-obtusin biosynthesis and transport. Higher concentration of the purine alkaloid in Guarana seeds was reported [79]. Higher aurantio-obtusin content in the seed also suggested, as in other higher plants containing secondary metabolites, that the aurantio-obtusin precursors could be involved in some protective functions [80]. With an aim to understand the genes leading to the aurantio-obtusin biosynthesis, we carried out a short-read NGS transcriptome of the *C. obtusifolia* seed previously [81]. Meanwhile, a few NGS transcriptome databases have been obtained from *Cassia angustifolia* [82,83] and *Cassia biscapsularis* [84] in the Caesalpiniaceae family, which belong to the Cassia genus. However, this short-read NGS study was limited by either number and/or length of the generated sequence information. In order to obtain full-length cDNA sequences, necessary further traditional gene cloning, which has been proven to be expensive, time-consuming, and inefficient, was required. The limited resources cannot provide more help to study *C. obtusifolia*.

Recently, the long-read SMRT sequencing was discovered for obtaining high percentage of full length cDNAs from the cDNA library. Moreover, the hybrid approach, combining both short-read NGS and long-read SMRT sequencing, led to the improvement of full length cDNAs of high quality, and thereby provided more accurately integrated analysis of transcriptomes than NGS. To date, hybrid transcriptome analysis followed by identification of potential candidate genes will lead to a better understanding of secondary metabolic pathways, the molecular mechanisms of organ formation and development, and the molecular mechanism of stress response as well in plants, such as *Astragalus membranaceus*, *Dendrobium officinale*, *Salvia miltiorrhiza*, *Triticum aestivum*, and Maize [18,85,86]. The transcriptome study of the pathways leading to production of natural products such as aurantio-obtusin will help to manipulate pathways in plants and reconstitute plant pathways in microbial hosts. In this study, the 108.13 Gb NGS data and 9.66 Gb SMRT data of various organs provided the first comprehensive insight into the various organs of *C. obtusifolia*, and might serve as the genetic background for *C. obtusifolia* to improve the basic biological research of *C. obtusifolia*. Interestingly, this is the first full-length transcriptome sequencing from plants in the Caesalpiniaceae family. More than 98.25% (57,092 of 58,106) nonredundant transcripts were annotated by sequence similarity search in public databases. The percentage of the annotated transcripts was far more than those in the former NGS data from *C. obtusifolia* (85%) [87], *Ligusticum chuanxiong* (61.54%) [16], and parsley (60.84%) [88]. Two genes with full-length sequences from transcriptome, *CoDXS* and *CoDXR*, were directly used to construct recombinant vectors, transferred into *E. coli* and proven to encode functional protein by color complementation assay. The results indicated that the full-length sequences were accurate and could be used directly for functional identification and further genetic manipulation. Hybrid transcriptome sequencing, as a high throughput as well as cost-effective approach of sequence determination, has dramatically improved the quality, efficiency, and speed of gene discovery.

*C. obtusifolia* is widely known for its pharmaceutical important anthraquinones and hence, gaining insights into the transcriptional regulation of anthraquinone biosynthesis in general could accelerate the engineering of this pathway for production of high anthraquinone content in the future. Using GO and KEGG annotation, we have identified large number of transcripts involved in metabolism, genetic information processing, environmental information processing, cellular processes, and organizational systems. All these transcripts are important resources for genetic manipulations of Cassia in the future. In general, anthraquinones from higher plants are derived through combination of isochorismate and MVA/MEP, and also the polyketide pathway. In the former pathway, the backbone of anthraquinones is synthesized via the isochorismate and MVA/MEP pathway. Most of the genes encoding enzymes involved in the isochorismate and MVA/MEP pathway were present in the transcriptome of *C. obtusifolia* in our study. There was more than one transcript assigned to the same enzyme. These results also demonstrated the powerful ability of high-throughput sequencing to identify genes in metabolic pathways. Such transcripts may represent different parts of a single gene, different members of a gene family, or both. Twenty-one (including DAHPS, DHQS, SDH, SMK, EPSP, CS, ICS, MenE, and MenB), seven (including HMGR, ACAT, and MPD), and 15 (including DXS, DXR, CDPMEK, ISPF, HDR, and IPPS) transcripts were found with FPKM > 10 in isochorimate, MVA, and MEP pathways, respectively. There were three rate limiting steps, which were catalyzed by IPPS, DXS, and ICS, respectively, in the early stage of anthraquinone formation [18,20,86]. Besides IPPS, DXS, and ICS, we also found that genes encoding DHQS, SDH, EPSP, ACAT, and MPD that showed the highest expression level in the *C. obtusifolia* seed, which suggested that these steps might be rate-limiting in the formation of dimethylallyl diphosphate leading to anthraquinone formation. These genes formed likely candidates for genetic manipulation of anthraquinone biosynthesis in *C. obtusifolia*. Functional characterization of the candidate genes will not only help elucidate the biochemical mechanism for life saving compounds biosynthesis, but also provide a molecular and biochemical target for improving the content of these compounds in future. Further enzyme assays of these enzymes are required to identify the function of the candidate genes. However, although three transcripts, including PKS III and PKC, were found with FPKM > 10 in the polyketide pathway in the seed, none of them showed the highest expression level in the seed, suggesting the anthraquinone might mainly be biosynthesized from isochorismate and MVA/MEP pathway in *C. obtusifolia*.

CYP450s, SAM-dependent methyltransferases, and UDPGs mainly medicated many modifications of the backbone of anthraquinones in the late stage of anthraquinone biosynthesis. In plants, CYPs catalyzed the addition of the oxygen atom to the metabolites and many of them were involved in plant secondary metabolism. SAM-dependent methyltransferases catalyzed the methylation of the hydroxyl group of substrate to generate methyl metabolites. UDPGs catalyzed glycosylation at the site of hydroxyl group to produce glycosylated metabolites. In total, 135, 92, and 82 transcripts in seed were identified as putative CYPs, SAM-dependent methyltransferases and UDPGs, respectively, using BLAST search. In the former seed transcriptome *C. obtusifolia* [18], only 30 CYPs, 12 SAM dependent methyltransferases, and 14 UDP-glucosyltransferase unigenes were identified [89], the number of these genes was far lower than those found in this study. These results also demonstrated the powerful ability of SMRT sequencing to identify genes in metabolic pathways. In present study, 21 CYPs, 17 SAM-dependent methyltransferases, and 25 UDPGs were found with FPKM > 10. Among them, nine CYPs, five SAM-dependent methyltransferases, and 16 UDPG exhibited the highest expression level in seed. Moreover, five CYPs, one SAM-dependent methyltransferase, and seven UDPGs were seed-specific. Therefore, further studies on higher expressed and seed-specific CYPs, SAM-dependent methyltransferases, and UDPGs genes are needed to better understand roles of these genes in the formation and development, and biosynthesis of anthraquinone in the seed; these genes could be applied to the genetic improvement of *C. obtusifolia* plants.

In addition, the potential function of several candidate CYPs was predicted by the polygenetic tree. The CYPs involved in brassinolide biosynthesis, including *Co*CYP450 Monooxygenase-1, *Co*CYP450-5 and 8, and monoterpenoid biosynthesis, including *Co*CYP450-10, 13, 16, and *Co*CYP450 reductase 1 were suggested to be promising candidates associated with anthraquinone biosynthesis. In the late stage of anthraquinone biosynthesis, the backbone was hydroxylated at 1C, 2C, 3C, 6C, 7C, and 8C positions to produce various anthraquinone compounds. Therefore, *Co*CYP450-10, 13, and 16 might be regarded as strong candidate CYP450s for the modification of anthraquinone backbone, and CYPs in group 2 are potential genes for further functional characterization.

Differentially (especially organ-specifically or tissue-specifically) expressed TFs might play an important role in recruiting nondifferentially expressed TFs to the TF–-TF interaction network, offering the potential for coordinating and controlling organ/tissue gene expression across a variety of conditions [90]. The 18 verified seed-specific TFs, especially the four TFs (F01.PB3829, 48100, 6578, and 409), provided regulatory network concerning the formation, development, secondary metabolism, and stress response of the seed. Interestingly, this was the first report that YABBY and HBP TFs expressed specifically in seed. Three MYB and one MADS TFs were suggested to be the strong candidate TFs involved in seed formation and development, according to Go annotation. Both MYB and MADS TFs known to regulate organ formation and development play important role in control of seed formation and development [91,92]. Meanwhile, alternative splicing might regulate seed formation and development at the post-transcriptional level. The alternative splicing took part in several biological processes, such as plant hormone signal transduction and sugar and carbon metabolism, and play an important role in barley seed germination [93]. To date, regardless of whether or not the above genes were involved with the seed formation and development needs further transgenic evidence, those expressing a high level in the seed indicated that they might play essential role in seed formation and development. The further qRT-PCR analysis showed that the gene expression profiles of TFs were consistent with those in comparative transcriptome analysis. These results indicated that it was effective to analyze the gene expression profiles in various organs by biological repeats in high-throughput sequencing technologies and qRT-PCR.

Previous research identified 51 Hsp20 genes in the genome of soybean; most members of which were induced by stresses [94]. The low number of Hsp20 genes in transcriptome of *C. obtusifolia* is due to the fact that it grew under normal condition. The expression patterns of Hsp20 genes in different tissues and organs have been described in Arabidopsis, rice, soybean, and potato [18,95,96]. There was no uniform gene expression pattern of plant Hsp20 genes, suggesting different Hsp20 proteins might have diversified functions in the growth, development, and stress response of the plant. According to the transcriptome data of *C. obtusifolia*, six genes, mostly *Co*Hsp20 genes (75%), displayed relatively higher expression levels in the seed and flower. Similar to several Hsp20 genes in *Panicum virgatum*, 60% members showed specifically expressed in seed and flower under normal condition [97]. Such expression profiles were also recorded with rice and Arabidopsis Hsp20s [98,99]. For example, eight Arabidopsis Hsp20 genes were specifically expressed in leaves, and some rice Hsp20s were specifically accumulated in seeds. Considering that the flower and seed are important reproductive organs, these relative high expression levels of *Co*Hsp20 genes in reproductive organs, even without stress, indicated that these *Co*Hsp20s played vital roles in maintaining cellular homeostasis during meiosis, fertilization, and seed setting. Several *Co*Hsp20 genes, such as *CoHsp3* and *CoHsp5*, exhibited quite different expression patterns in various organs, indicating that different *Co*Hsp proteins may have diverse functions. Furthermore, qRT-PCR was used to investigate the transcript levels of each *Co*Hsp20 under different abiotic stresses. *Co*Hsp20*-6* with high expression levels in the seed was not induced under various stresses. Thus, we might assume that *Co*Hsp20*-6* is lacking of chaperone activity. Similar expression patterns in five genes (*Co*Hsp20-*1*, *3*, *4*, *7*, and *8*) under heat stress might be caused by shared induction mechanisms. It was reported that the expression of Hsp20 family was controlled by shock transcription factors [100], thus the similar expression pattern of these five *Co*Hsp20 genes under heat stress might be attributed the similar upstream regulating genes of heat shock transcription factors. Moreover, *Co*Hsp20*-1*, *4*, *7*, and *8* were upregulated under other abiotic stresses; however, *Co*Hsp20*-3* was not sensitive to other abiotic stresses. The differences might be due to the fact that various *cis*-elements existed in promoter regions of different *Co*Hsp20 genes that were involved in the responses of *Co*Hsp20 genes to other abiotic stresses. Therefore, the various responses of *Co*Hsp20 genes reflected a complicated interconnected induction mechanism involving both *cis*-elements and heat shock transcription factors.

## 4. Methods and Materials

### 4.1. Plant Materials

The *C. obtusifolia* was cultivated in a controlled environment growth chamber under a 16 h/8 h photoperiod at 25 °C/16 °C day/night cycle. The seed, root, stem, flower, and leaf of *C. obtusifolia* 40 days after flowering (DAF) were collected. All the organs were immediately frozen in liquid nitrogen and stored at −80 °C for RNA isolation in the future. Fifteen total RNA samples (five different organs with three repetitions) were isolated using the RNeasy Plus Mini Kit (Qiagen Corporation, Hilden, Germany). The total RNA samples were treated with Dnase I to remove DNA contaminant. RNA quantity and quality were determined using Nanodrop, gel electrophoresis, and further by the Agilent 2100 Bioanailzer (Agilent, Santa Clara, CA, USA). 

### 4.2. Determination the Content of Aurantio-Obtusin 

In order to determine the content of aurantio-obtusin, the seed, root, leaf, flower, and stem of *C. obtusifolia* were firstly extracted, respectively, by means of reflux extraction using methanol as solvent. Secondly, the extractions were centrifuged at 4 °C, 10,000 rpm, 15 min and dried by distillation. Then, the residual material was hydrolyzed by hydrochloric acid and extracted with chloroform, and dried by distillation. Moreover, the dried material was dissoluted by anhydrous ethanol-ethyl acetate (2:1, volume ratio) and filtered through 0.45 μm microporous filter membrane. Finally, the content of aurantio-obtusin was determined by HPLC using Hypersil ODS2 column (4.6 mm × 200 mm, 5 μm) with flow rate of 1 mL min^−1^, detection wavelength at 284 nm, temperature at 30 °C, acetonitrile, and 0.1% phosphoric acid in water as mobile phase. 

### 4.3. Illumina Library Construction and Sequencing

The Poly (A) mRNA from fifteen samples of five various organs (three repetitions with each organs) was enriched from total RNA using oligo (dT) magnetic beads. Following the enrichment, the mRNA was fragmented into small pieces within fragmentation buffer. Using these short fragments as templates, the first-stand cDNA was synthesized using Superscript™ III reverse transcriptase and random hexamer (N6) primers. Subsequently, the RNA templates were removed and the second-strand cDNA was synthesized using dNTPs, DNA polymerase I, and RNase H. These short double cDNA fragments were purified with AMPure XP beads. After end reparation and A-tailing, the short cDNA fragments were ligated with the Illumina paired-end adaptors and purified with AMPure XP beads. Then, PCR was used to selectively enrich DNA fragments with adapter molecules on both ends and to create the final cDNA library. The concentration of the cDNA library was assessed using Qubit 2.0 fluorometer (Life Technologies, Carlsbad, CA, USA) and the quality of the cDNA library was measured using the Agilent 2100 Bioanalyzer. Finally, the 15 libraries were sequenced from both the 5′ and 3′ ends using Illumina HiSeq™ 4000 system (Illumina, San Diego, CA, USA), respectively.

### 4.4. Single-Molecule Real-Time Library Construction and Sequencing

Total RNA of five individual samples (seed, root, stem, leaf, and flower) was pooled to provide 42 μg of total *C. obtusifolia* RNA. Poly (A) RNA was isolated from the total RNA using the oligo (dT) magnetic bead binding method and the Poly (A) PuristTM Kit. Isolated poly (A) RNA was eluted with 20 μL of RNase-free water. One microgram of RNA was reversely transcribed using Clontech SMARTer cDNA synthesis kit. After PCR amplification, quality control, and purification, we performed size selection using BluePippin Size Selection System protocol and herein produced three fractions containing fragments of 1–2, 2–3, and 3–6 kb in length, respectively. The cDNA products were then subjected to construction of SMRTbell Template libraries using SMRTBell Template Prep Kit. The concentration and quality of the cDNA library were measured using Qubit 2.0 fluorometer and Agilent 2100 Bioanalyzer, respectively. Finally, a total of four SMRT cells were sequenced on PacBio RS II platform (Pacific Biosciences, Menlo Park, CA, USA).

### 4.5. Error Correction of PacBio Reads

According to the official protocol, raw polymerase reads that had full passes ≥0 and the predicted consensus accuracy >0.75 were selected for producing ROIs. After the ROIs shorter than 50 bp in length were discarded, they were classified into full-length (FL) and non-full-length (nFL) reads according to whether 5′/3′ cDNA primers and poly (A) tail were simultaneously observed or not. We subsequently employed three strategies for improving accuracy of PacBio reads. First, all sequences were subjected to isoform-level clustering by Iterative isoform-clustering (IEC) algorithm and herein produced consensus sequences of isoform. Second, the isoform sequences were corrected to obtain high-quality isoforms and low-quality isoforms using Quiver software. Third, the low-quality isoforms were corrected in aid of Illumina short reads using Proovread tool with default setting. Finally, the longest isoform of each cluster was regarded as the transcript which was followed with correction by CD-HIT software to remove redundant information for further functional annotation. 

### 4.6. Functional Annotation and Classification

In order to predict putative gene function, transcripts were aligned with the NR (available online: http://www.ncbi.nlm.nih.gov/), SwissProt (available online: http://www.uniprot.org/), GO (available online: http://www.geneontology.org/), COG (available online: http://www.ncbi.nlm.nih.gov/COG/), and KEGG (available online: http://www.genome.jp/kegg/) databases using BLASTX with E-value of 1 × 10^−5^. When the results from different databases conflicted, a priority order of NR, Swiss-Prot, KEGG, and COG was obeyed. Then, based on the best BLAST hits from the KEGG database, KEGG orthology was performed using KOBAS software 2.0. At last, the amino acid sequences of all the transcripts were annotated using HMMER software (E-value < 1 × 10^−10^) with the PFAM (available online: http://pfam.xfam.org/) to produce the final annotation information of transcripts.

### 4.7. Determination of Relative Gene Expression Level

Relative gene expression levels of each transcript was determined by calculating the sum of the fragments mapping to each transcript, and then normalized by converting the fragments counts to (fragments per kilobase of transcript per million mapped reads, or FPKM) across the transcriptome.

Differential expression analysis was performed using DESeq [101]. Those genes having a fold change ratio ≥ 2 and 5, with corrected *p* value < 0.01 were considered as differentially expressed genes and specially expressed genes, respectively [9,102]. The transcriptome analysis pipeline and strategy involved in the present study was outlined in Figure S1. 

### 4.8. De Novo Detection and Validation of Alternative Splicing Events

The alternative splicing (AS) events were obtained according to the method of Liu et al. [103]. The prediction principle is based on pairwise alignment using BLAST. If the alignment result of two isoforms satisfy the following criterions, both are considered to be from one AS event. First, both isoforms are longer than 1000 bp and have two HSPs (High-scoring Segment pairs). Second, the AS GAP is no less than 100 bp and is at least 100 bp away from the 3′ and 5′ end. Third, the overlap between variable transcripts is no more than 5 bp. 

### 4.9. Long Noncoding RNA Identification

To define a set of putative lncRNAs in the transcriptomes of *C. obtusifolia*, we applied several filtering criteria. All of the transcripts not overlapping with *C. obtusifolia* protein-coding genes and being located at least 1 kb away from the closest annotated protein were considered for our analysis. A series of filtering steps were then implemented. The first one consisted of selecting the transcripts for which Coding Potential Calculator (CPC) [104] returned no (or just partial) coding potential. Second, the Coding–Noncoding Index (CNCI) [105] was then used to search all possible protein noncoding sequences. Similarly, the Coding Potential Assessment Tool (CPAT) was used to search the possible translational products based on the length and coverage of ORF [106]. Finally, the Hmmscan tool was used to search the possible translational products of each transcript against a database of Pfam profiles and the transcripts returning an expectation value lower than 1 × 10^−3^ were removed. 

### 4.10. Phylogenetic Analysis

In order to identify candidate CYPs involved in the aurantio-obtusin biosynthesis, the seed-specific CYPs with sequence length over 200 amino acids were selected and then aligned with 61 CYPs from various plants using MEGA 6.0 (Arizona State University, Tempe, AZ, USA). The unrooted phylogenetic tree was constructed using the neighbor-joining clustering method with bootstrap values obtained after 1000 replications.

### 4.11. Functional Analysis of CoDXS and CoDXR Genes

In order to validate the accuracy of the sequences obtained from the hybrid platform, two full-length transcripts (F01.PB17587 and F01.PB29801) encoding *C. obtusifolia* 1-deoxy-d-xylulose 5-phosphate synthase (*CoDXS*) and 1-deoxy-d-xylulose 5-phosphate reductoisomerase (*CoDXR*) were directly chosen for functional analysis, respectively, using bacterial complementation assay [107]. In higher plant, DXS and DXR played an important role in the MEP pathway and were involved in the biosynthesis of anthraquinone [108]. The plasmids pAC-BETA and pTrc-AtIPI were purchased from ADDGENE Corporation (USA). The pAC-BETA contained all functional genes of the *β*-carotene synthesis, and also retained a chloramphenicol (Chl) resistance gene. Cells of *E. coli* containing pAC-BETA produced and accumulated *β*-carotene, resulting in yellow colonies. The pTrc-AtIPI contained a Trc promoter, ampicillin resistance gene and *AtIPI* gene. 

The ORF of *CoDXS* was amplified by PCR with forward primer (5′-A TAA GAA TGC GGC CGC *ATG* GCT CTT TGC ACA TTC TC-3′, the *Not*1 site is underlined and the initiation codon is shown in italics) and reverse primer (5′-CGC GGA TCC *TTA* TGA CAA AAC CTC TAA TGC CTC-3′, the *Bam*H1 site is underlined and the stop codon is shown in italics). The coding region of *CoDXR* gene was amplified by PCR using the forward primer (5′-CG GAA TTC *ATG* GCT CTG AAT TTG 3′, the *Eco*R1 site is underlined and the initiation codon is shown in italics) and the reverse primer (5′-GA AGA TCT *TCA* TGC AGG AAT AGG A-3, the BglII site is underlined and the stop codon is shown in italics). The fragments were cloned into a pMD19-T vector for sequencing to confirm that no substitutions or deletions occurred. After digestion of PCR fragments and plasmid pTrc-AtIPI with corresponding endonucleases, the recombinant plasmid pTrc-*Co*DXS and pTrc-*Co*DXR were generated and subsequently verified by sequencing, respectively.

The pTrc-AtIPI digested by PstI to remove *AtIPI* and then ligated by T4 DNA ligase was used as control. The plasmids pTrc-*Co*DXS and pAC-BETA, as well as the plasmids pTrc-*Co*DXR and pAC-BETA, were cotransformed into *E. coli* Top10, respectively. The plasmids pTrc and pAC-BETA were also cotransformed and the single plasmid pAC-BETA, which was transformed into the *E. coli* Top10 to be used as the negative controls. Transformants were cultured on solid LB medium containing ampicillin (150 mg/L) and Chl (50 mg/L) at 37 °C in dark for 2 days.

### 4.12. Quantitative Real-Time PCR (qRT-PCR) Assay

To analyze the expression levels of mRNA of potential genes and TFs related to anthraquinone biosynthesis, seed formation and development, and stress response in various organs of *C. obtusifolia*, the total RNA of various organs were extracted and subjected to cDNA synthesis and further qRT-PCR, respectively. Moreover, the expression pattern of 8 Hsp20 genes under heat, chilling, drought, and salt stresses was detected. For chilling and heat stress, *C. obtusifolia* were cultivated at 4 and 37 °C for 0, 1, 6, 12, 24, and 48 h, respectively. For drought and salt stresses, *C. obtusifolia* were cultivated supplemented with PEG 6000 (100 mg mL^−1^), or NaCl (200 mM) for 0, 1, 6, 12, 24, and 48 h, respectively. The qRT-PCR was performed using SYBR Premix Ex TaqTM II (Takara, Japan) with a LightCycler 96 system (Roche Diagnostics, Mannheim, Germany). The total RNA of *C. obtusifolia* leaf under different stresses were extracted and subjected to cDNA synthesis, respectively. 

In each run, 1 μL cDNA template was added to 10 μL reaction buffer using gene-specific primer pairs under the following conditions: initial denaturation at 95 °C for 30 s, 40 cycles of 5 s at 95 °C, 10 s at 60 °C, and 25 s at 72 °C. All samples were performed in triplicate. Then, based on the transcriptome data, the seed-specific CYPs, SAM-methyltransferases, UDPGs, TFs, and 8 Hsp20s were selected for verification by qRT-PCR using *EF1α* as a reference gene [109]. The relative expression levels of the selected genes were normalized to the selected reference gene and determined by the ΔΔ*C*t-method [18]. All the Primer sequences used in this study were listed in Table S1. 

## 5. Conclusions

Compared with previous traditional cDNA cloning or NGS studies in *C. obtusifolia*, we produced a more comprehensive transcriptome data set with the following features. First, many longer (81.73%, >1000 bp) sequences with high quality and more annotation ratio (98.25%) were generated in our hybrid sequencing platform, providing a direct use for further genetic functional studies without additional RACE to get a complete ORF. Second, more sequences (1214) were involved in secondary metabolism and more sequences (607) involved in the anthraquinone backbone biosynthesis were found in the hybrid platform, whereas only 455 and 185 sequences were found in previous NGS data, respectively. Moreover, the hybrid platform provided an opportunity to detect alternative splicing and the 2712 candidate AS events suggesting a high degree of transcriptome complexity in *C. obtusifolia*. Although aurantio-obtusin biosynthesis-related genes (all with FPKM > 10 in seed) and Hsp20 genes do not seem to undergo any significant degree of alternative splicing, several potential TFs involved in the seed formation and development were identified as undergoing alternative splicing in *C. obtusfiolia* (Figure S3). It should be noted that only one isoform was predominant (with FPKM > 10 in seed), suggesting that another alternative isoform may not be significant. Nevertheless, alternative splicing is clearly observed among the TFs involved in seed formation and development (Table 3), which may serve as a regulatory mechanism in controlling seed formation and development. Further studies are required to investigate the biological functions of these transcript isoforms. Although only a minority of lncRNAs could be annotated with known RNA motifs, these still provide a useful resource for understanding the potential functions of lncRNAs in *C. obtusifolia*. Finally, we characterized the strong candidate genes in the biosynthesis of aurantio-obtusin from *C. obtusifolia*, TFs involved in the seed formation and development, and stress-responsive *Co*Hsp20 genes as well.

## Figures and Tables

**Table 1:** PacBio libraries and sequencing results.

cDNA Size	Number of Consensus Isoforms	Average Consensus Isoforms Read Length	Number of Polished High-Quality Isoforms	Number of Polished Low-Quality Isoforms	Percent of Polished High-Quality Isoforms (%)
0–1 kb	6206	897	5729	477	92.31%
1–2 kb	25,179	1371	21,937	3242	87.12%
2–3 kb	17,457	2457	14,323	3134	82.05%
3–6 kb	17,616	3485	12,537	5079	71.17%
>6 kb	764	9064	7	757	0.92%
All	67,222	2250	54,533	12,689	81.12%

**Table 2:** Summary of database matches for the *C. obtusifolia.*

Anno Database	Annotated Number	300 ≤ Length < 1000	Length ≥ 1000
COG_Annotation	24,630	1706	22,924
GO_Annotation	40,502	3344	37,158
KEGG_Annotation	25,573	2147	23,426
KOG_Annotation	36,919	2704	34,215
Pfam_Annotation	47,734	3576	44,158
Swissprot_Annotation	43,158	3383	39,775
eggNOG_Annotation	56,156	4598	51,558
Nr_Annotation	56,681	4767	51,914
All_Annotated	57,092	4800	52,292

**Table 3:** The transcripts related to secondary metabolites.

Biosynthesis of Secondary Metabolites	Transcript Numbers
Brassinosteroid biosynthesis	8
Caffeine metabolism	14
Carotenoid biosynthesis	130
Diterpenoid biosynthesis	37
Flavone and flavonol biosynthesis	6
Flavonoid biosynthesis	177
Isoquinoline alkaloid biosynthesis	51
Limonene and pinene degradation	28
Monoterpenoid biosynthesis	5
Phenylpropanoid biosynthesis	363
Stilbenoid, diarylheptanoid, and gingerol biosynthesis	52
Terpenoid backbone biosynthesis	165
Tropane, piperidine, and pyridine alkaloid biosynthesis	85
Steroid biosynthesis	79
Zeatin biosynthesis	14
Total	1214

## Supplementary Materials

The following are available online at http://www.mdpi.com/1422-0067/19/9/2476/s1.

## Abbreviations

*C. obtusifolia*	*Cassia obtusifolia*
*Co*	*Cassia obtusifolia*
TFs	Transcription Factor
CYPs	Cytochrome p450/Cytochrome p450 Monooxygenase/NADPH-cytochrome p450 reductase
*Co*CYP450	*Cassia obtusifolia* Cytochrome p450
*Co*Hsp20	*Cassia obtusifolia* heat shock protein
*Co*Reductase	*Cassia obtusifolia* Cytochrome p450 reductase
*Co*ARF	*Cassia obtusifolia* Auxin-Responsive Transcription Factor
*Co*LEA	*Cassia obtusifolia* Late Embryogenesis Abundant Transcription Factor
*Co*NF	*Cassia obtusifolia* Nuclear Transcription Factor
*Co*HBP	*Cassia obtusifolia* HBP Transcription Factor
*Co*MADS	*Cassia obtusifolia* MADS-box Transcription Factor
*Co*MYB	*Cassia obtusifolia* MYB Transcription Factor
*Co*NAC	*Cassia obtusifolia* Nac Transcription Factor
*Co*bHLH	*Cassia obtusifolia* bHLH Transcription Factor
*Co*WRKY	*Cassia obtusifolia* WRKY Transcription Factor
*Co*YABBY	*Cassia obtusifolia* YABBY Transcription Factor
*Co*ZF	*Cassia obtusifolia* Zinc Finger Transcription Factor
*Co*SAMT	*Cassia obtusifolia* di-*O*-methyltransferase
GATA	GATA transcription factor
Trihelix TFs	Trihelix Transcription Factor
ACAT	Acetyl-CoA C-acetyltansferase
HMGS	Hydroxymethylglutaryl-CoA synthase
HMGR	Hydroxymethylglutaryl-CoA reductase
MK	Mevalonate kinase
PMK	Phosphomevalonate kinase
MPD	Diphosphomevalonate decarboxylase
DXS	1-deoxy-d-xylulose-5-phosphate synthase
DXR	1-deoxy-d-xylulose-5-phosphate reductoisomerase
ISPD	1-C-methyl-d-erythritol 4-phosphate cytidylyltransferase
CDPMEK	4-diphosphocytidyl-2-C-methyl-d-erythritol kinase
ISPF	1-C-methyl-D-erythritol 2,4-cyclodiphosphate Synthase
HDS	(E)-4-hydroxy-3-methylbut-2-enyl-diphosphase synthase
HDR	4-hydroxy-3-methylbut-2-enyl-diphosphase reductase
IPPS	Isopentenyl-diphosphate delta-isomerase
DAHPS	3-deoxy-7-phosphoheptulonate synthase
DHQS	3-dehydroquinate synthase
SDH	3-dehydroquinate dehydratase
DHQD	Shikimate dehydrogenase
SMK	Shikimate kinase
EPSP synthase	3-phosphoshikimate 1-carboxyvinyltransferase
CS	Chorismate synthase
ICS	Menaquinone-specific isochorismate synthase
menC	2-succiny-6-hydroxy-2,4-cyclohexadiene-1-carboxylate synthase
PHYLLO	*O*-succinyibenzoate synthase
MenH	2-succinyl-6-hydroxy-2,4-cyclohexadiene-1-carboxylate synthase
menD	2-succinyl-5-enolpyruvyl-6-hydroxy-3-cyclohexene-1-carboxylate synthase
menE	Acyl-activating enzyme
menB	Naphthoate synthase
PSKIII	Polyketide synthaseIII
PKC	Polyketide cyclase/dehydratase
UDPG	UDP-Glucosyl transferase
