# Supplementary material for: Full-Length Transcriptome Survey and Expression Analysis of Cassia obtusifolia to Discover Putative Genes Related to Aurantio-Obtusin Biosynthesis, Seed Formation and Development, and Stress Response
Source: Int J Mol Sci. 2018 Aug 21;19(9):2476. doi: 10.3390/ijms19092476 (PMC6163539; doi:10.3390/ijms19092476)
Supplement: Supplementary file 1 [file ijms-19-02476-s001.pdf]

Supplementary Materials: Full-Length Transcriptome Survey and Expression Analysis of *Cassia obtusifolia* to Discover Putative Genes Related to Aurantio-Obtusin Biosynthesis, Seed Formation and Development, and Stress Response

Yin Deng, Hui Zheng, Zicheng Yan, Dongying Liao, Chaolin Li, Jiayu Zhou and Hai Liao

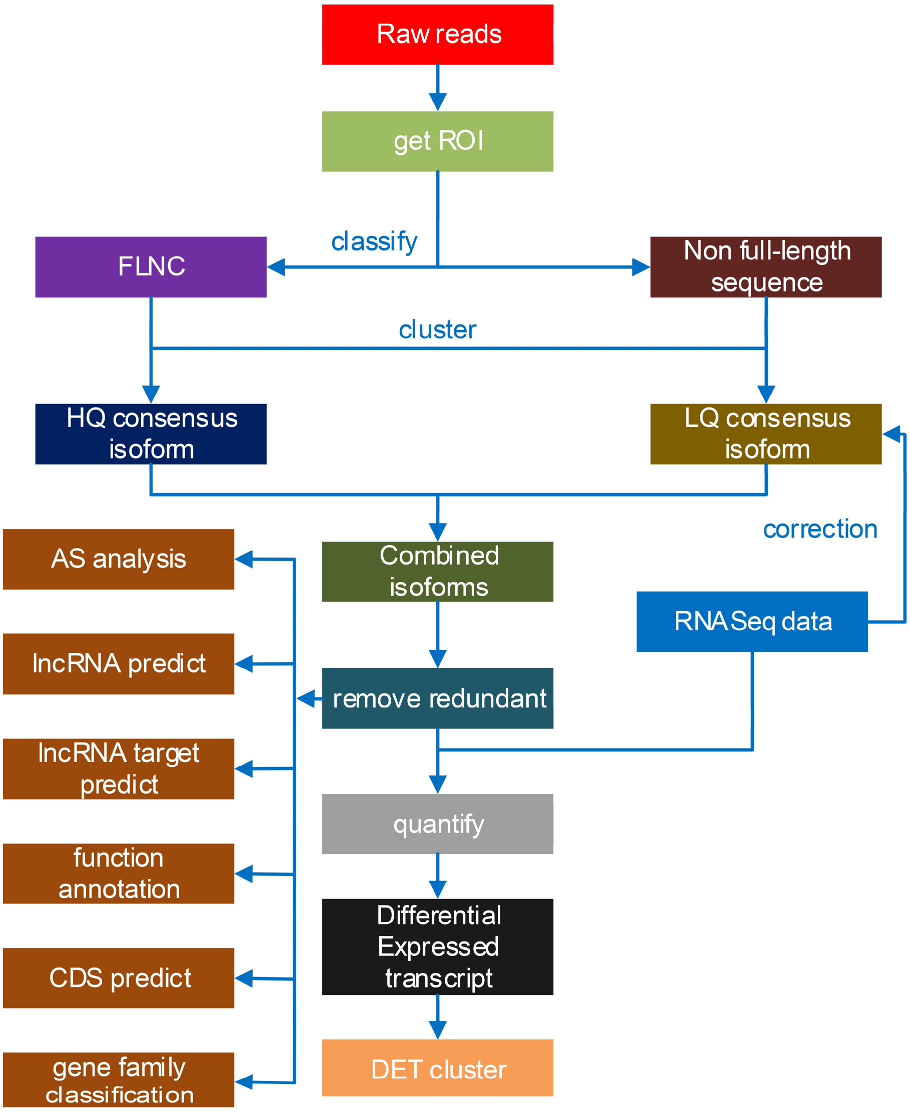

Figure S1. Transcriptome analysis pipeline and strategy used for the hybrid transcriptome assembly and characterization.

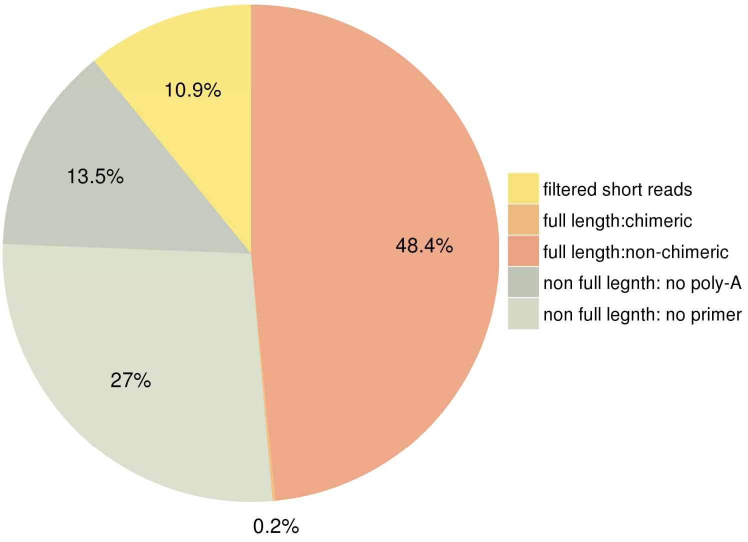

Figure S2. Classification of ROIs.

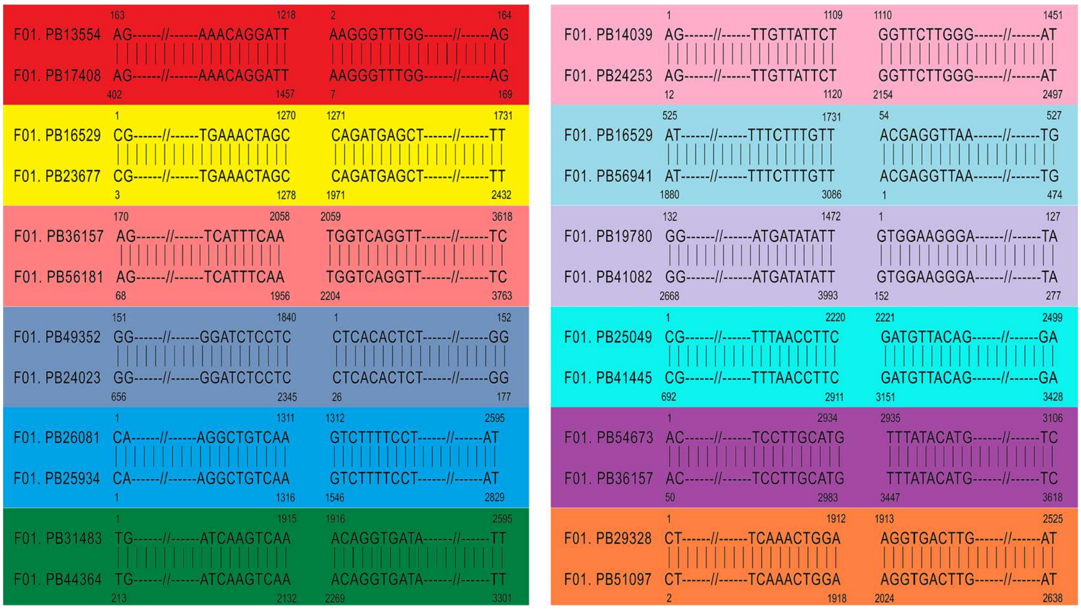

Figure S3. The different alternative splicing isoforms of transcriptional factor genes involved in seed formation and development.

Table S1. Primers used in the study.

Primer	sequence
Oligo(dT)18	GCTGTCAACGATACGCTACGTAACGGCATGACAGTGTTTTTTT TTTTTTTTTTT
EF1α2qFP	TTGAAGAACGGTGATGCAGGTA
EF1α2qFP	CACACTCTTGATGACTCCCACA
CoHSP20-1qFP	TTCGACCTTTCTCTCTCGACC
CoHSP20-1qRP	CTCCTCGATCTCCACCTTC
CoHSP20-2qFP	GCTATGCCACAACCAGATGAG
CoHSP20-2qRP	CTGCACTGATGGTGGTGTC
CoHSP20-3qFP	TGCCTTATCGCTTCTACTTCTC
CoHSP20-3qRP	CCTCTAGAGGAAGACGATGATC
CoHSP20-4qFP	CGGTGGTAGAAGGAGCAAC
CoHSP20-4qRP	GGAGCACCCTGTCATCCTC
CoHSP20-5qFP	CAGACAATCCTACACAGTTCCTC
CoHSP20-5qRP	GGTGGAATGGAATCTTGGGC
CoHSP20-6qFP	CCACACACTCTTCTCCTTCTC
CoHSP20-6qRP	CCCAGAGAGATCGCGTGTAG
CoHSP20-7qFP	CTCCGTCAATATCAATGGCTCTC
CoHSP20-7qRP	GAACGCATCTGCCACTGAG
CoHSP20-8qFP	GACTGAGGCAGTGAACCAC
CoHSP20-8qRP	CACGGTCACCTGAATGTCAG
CoUDPG1qFP	CTCTATCTGCTCTGCTCTCCG
CoUDPG1qRP	ACACTTGCTCTGTTCTGAACC
CoUDPG2qFP	GTTCTGAGGACTCAGCGTG
CoUDPG2qRP	AGATCCATCTCGCTCGTCC
CoUDPG3qFP	ACCGTAGCATTCCGTTATTTCG
CoUDPG3qRP	TGTCGTTCTTCTCCTCGTCG
CoUDPG4qFP	CGCGGAATAGTGTGGAACAG
CoUDPG4qRP	GCTTCAACGACGTCGTCAC
CoUDPG5qFP	CGATCTGCCTTGCTACGTTTAC
CoUDPG5qRP	CCTATGGAGTTGCTTGCTCAC
CoUDPG6qFP	GCGGCTATACAGAACTGCG
CoUDPG6qRP	GCTTCTCCAACCAATCCTTCAC
CoUDPG7qFP	GCTCATGATCTCTTGGATGCC

CoUDPG7qRP	GGTTGGTTATCCAACCACGAC
CoCYP450-1qFP	GACACCACATCCACACTGC
CoCYP450-1qRP	TCACTATCACATGTGTTCCTGC
CoCYP450-2qFP	ACGAGAATCCATGCAAGACATC
CoCYP450-2qRP	TCAAAGTAGTGCACAAGGTTGG
CoCYP450-3qFP	CTTGCAAGTGAATCTGAGTGAGG
CoCYP450-3qRP	GATCCAACTCTTGAGCTACTCTG
CoCYP450-4qFP	TGTGTCAAGATGTGTGCTTGG
CoCYP450-4qRP	TCACATCATCAAAGACAGCATCC
CoCYP450-5qFP	GGTTCTGAGGCTAAGACCATG
CoCYP450-5qRP	GCAGCTCTCATTGCACAATG
CoDXSqFP	CATGTAGTTGCTGTTATTGGCG
CoDXSqRP	CCACTGATCATACCACGAGC
CoIPPSqFP	TTCAGCAACGATCTGCAACG
CoIPPSqRP	GCGAACATTAACATCCCGGAC
CoMDTqFP	CCTTACATGCTCATGATCTCTCG
CoMDTqRP	TTGTGATAGTGTTCCACCGC
CoICSqFP	ATTGCAAGTCGCTTGGAGC
CoICSqRP	ATTGTGTTGATCATTGCCTCGG
CoARFqFP	GTACAGCCAGATGCTATGTGG
CoARFqRp	CCAGATTCTGGTGATTATGCGC
CoLEA1qFP	CGCATTATAACCGAAGCAGTCG
CoLEA1qRP	TGTTATCACATTGCTTCCCGTC
CoLEA2qFP	GAGAATACTGCCGCAGCTAATG
CoLEA2qRP	TCCTCGTCTTCATCATGCTGG
CoLEA3qFP	GTGATGCAATCTGCAGCGTC
CoLEA3qRP	GCTTCTCCTATGGTTATGGCATC
CoNFqFP	GCATCATGCTAGAATGCCATTG
CoNFqRP	TTCAGAGGTAAGCAGGCTGC
CoHBPqFP	GCTCATCGACAAGATGATGTCAC
CoHBPqRP	GATTCAGTGTAGACGAGGTGG
CoMADS1qFP	CGTGGAAGAATGCACAATGAG
CoMADS1qRP	GATATTCAGCCTCACACAGCG
CoMADS2qFP	CAGTCGTGGTCGTCTCTATGAG
CoMADS2qRP	AGTGAGTGTGCTCAAGGCATC

CoMADS3qFP	CCTAAGACATGTGCAGCTGC
CoMADS3qRP	CACTACTGCCATCGTCTCTG
CoMYP1qFP	GAATCTCAGCCTCTCCACTGG
CoMYP1qRP	ATGGCACATCTCTCTTCTCATC
CoMYP2qFP	CTGATATGCTGGCTTGCTTCTC
CoMYP2qRP	GGTTGGCAGAATAGAATGGAGC
CoMYP3qFP	CTCAGATTGCAGCTCAGTTACC
CoMYP3qRP	GGAGAAGCAAGCCAGCATATC
CoNACqFP	CTCGTGATGCACTATCTCTGC
CoNACqRP	GCTTATCTGCACCAGTTGCC
CobHLHqFP	GCAGCCAGACGAAGAACAAC
CobHLHqRP	GATCTGAGAACTCCGAGGTGG
CoWRKYqFP	TGAGGACATCTGCATCTGACG
CoWRKYqRP	CTACAGTAGGTTCATTGGATGGC
CoYABBY1qFP	TGATACTGTCCTCGCTGTAAGC
CoYABBY1qRP	GGCAGTCCTAGATGGCATACAG
CoYABBY2qFP	CATCATCCTCATCAACTACTGCC
CoYABBY2qRP	CATCGCACAGTCACTGTCTTG
CoZF1qFP	GTCATGACTGGACGGACTGC
CoZF1qRP	GAGGAGTGTGAGCGAAGAAGC
CoZF2qFP	GTCGTCTAGTGCGTTGATGAGC
CoZF2qRP	GCTGCTGCTGATGATGATGATC

Table S2. The putative genes (all with FPKM>10 in seed) related to anthraquinone biosynthesis, seed formation and development, and stress response.

gene ID	Expression in root	Expression in stem	Expression in leaf	Expression in flower	Expression in seed	Annotation
F01.PB10020	34.28	21.85	8.31	49.55	285.93	Udp-Glycosyltransferase 91A1
F01.PB10650	22.50	35.32	37.33	11.48	14.38	1-Deoxy-D-Xylulose-5-Phosphate Synthase
F01.PB10833	0.00	0.31	0.00	2.20	59.35	Udp-Glycosyltransferase 91A1
F01.PB10944	22.93	6.72	0.37	92.22	110.58	Udp-Glycosyltransferase
F01.PB11031	17.92	15.02	4.77	24.54	10.20	Isopentenyl-diphosphate Delta-isomerase II
F01.PB11223	19.52	16.62	9.75	7.62	10.13	Shikimate Kinase
F01.PB11533	30.65	29.44	0.01	46.00	14.55	Cytochrome P450 71A1
F01.PB11581	3.37	1.70	36.68	27.68	34.27	Cytochrome P450 85A
F01.PB11939	7.86	5.22	11.67	2.37	10.95	Udp-Glucoronosyl/Udp-Glucosyl Transferase Family Protein
F01.PB12489	32.67	103.54	248.65	118.43	35.50	4-Hydroxy-3-Methylbut-2-Enyl Diphosphate Reductase

F01.PB1265	30.66	51.48	32.88	12.84	10.50	Chorismate Synthase 1
F01.PB13542	19.44	3.64	3.37	48.45	16.24	Cytochrome P450 734A1
F01.PB13551	15.24	20.43	31.09	34.09	37.09	3-Dehydroquinate Synthase
F01.PB14067	12.09	13.99	8.41	16.74	14.78	Diphosphomevalonate Decarboxylase
F01.PB14731	68.56	29.34	46.03	19.58	17.25	Acyl-Activating Enzyme 5
F01.PB14944	37.50	24.56	12.94	13.22	14.30	3-Phosphoshikimate 1-Carboxyvinyltransferase
F01.PB15276	0.11	1.27	0.11	0.26	21.68	Cytochrome P450 Family 71 Protein
F01.PB15287	3.61	10.32	0.17	2.75	70.82	Cytochrome P450 716B2
F01.PB15419	8.82	6.12	0.00	1.92	19.83	Cytochrome P450 Family 71 Protein
F01.PB15474	10.34	15.82	14.17	89.86	10.49	Cytochrome P450 86B1
F01.PB16224	66.69	42.64	79.16	91.69	31.93	Cytochrome P450 71A24
F01.PB16738	10.35	6.18	0.16	2.80	18.65	Acetyl-Coenzyme A Carboxylase Carboxyl Transferase Subunit Alpha
F01.PB16761	9.26	11.91	14.08	15.15	14.59	1-Deoxy-D-Xylulose-5-Phosphate Synthase
F01.PB17172	7.93	0.02	0.06	0.20	18.55	1-Deoxy-D-Xylulose-5-Phosphate Synthase 2

F01.PB17233	33.97	43.65	26.52	64.85	72.33	Isopentenyl-Diphosphate Delta-IsomeraseII
F01.PB1750	8.15	21.20	0.29	3.28	17.99	1-Deoxy-D-Xylulose 5-Phosphate Synthase
F01.PB17587	22.42	31.21	45.16	25.01	11.34	1-Deoxy-D-Xylulose 5-Phosphate Reductoisomerase
F01.PB17961	15.06	13.94	36.50	17.10	11.04	Type III Polyketide Synthase
F01.PB18404	67.74	75.63	41.94	61.54	59.36	Peroxisomal Enoyl-Coa Hydratase/Isomerase Family Protein
F01.PB18582	4.01	73.43	68.29	12.26	43.29	Limonoid Udp-Glucosyltransferase
F01.PB1877	1.90	14.40	0.03	12.02	193.49	Hypothetical Protein Prupe_Ppa010752Mg
F01.PB19520	97.37	22.59	83.83	16.56	27.10	Cytochrome P450 Family 709 Protein
F01.PB19914	7.83	9.93	9.12	13.30	13.90	Mevalonate Diphosphate Decarboxylase
F01.PB20158	4.32	27.18	86.47	8.95	15.78	Cytochrome P450 98A2
F01.PB2045	40.45	20.16	6.01	18.17	18.17	Isopentenyl Diphosphate Isomerase
F01.PB20786	1.65	17.90	66.99	52.29	25.41	Cytochrome P450 Family

						71 Protein
F01.PB21451	10.33	13.63	21.75	21.47	10.20	4-Diphosphocytidyl-2-C-Methyl-D-Erythritol Kinase
F01.PB22057	1.65	1.22	0.00	66.91	173.63	Udp-Glucosyltransferase Family Protein
F01.PB22220	1.13	3.07	6.90	9.90	12.90	3-Dehydroquinate Synthase
F01.PB22595	5.34	5.45	5.79	14.48	10.82	Cytochrome P450 Monooxygenase Cyp51G1
F01.PB22877	1.39	0.80	0.05	129.09	536.97	Udp-Glucosyltransferase Family Protein
F01.PB23040	14.74	39.73	145.53	52.37	32.94	Polyketide Cyclase/Dehydrase And Lipid Transport Superfamily Protein
F01.PB23612	12.28	44.69	18.59	135.98	41.53	3-Hydroxy-3-Methylglutaryl-Coenzyme A Reductase
F01.PB24506	9.52	15.41	20.96	12.72	13.82	Nadph--Cytochrome P450 Reductase
F01.PB24938	36.15	31.92	43.68	35.56	53.32	3-Phosphoshikimate 1-Carboxyvinyltransferase
F01.PB25321	3.65	6.54	24.06	16.34	20.70	Phospho-2-Dehydro-3-Deoxyheptonate Aldolase 2

F01.PB25462	1.66	4.07	8.44	5.63	12.39	Cytochrome P450 97B2
F01.PB2613	0.46	13.12	93.96	18.50	20.22	Cytochrome P450 89A2
F01.PB28214	6.42	3.49	8.34	6.95	14.74	Acyl-Activating Enzyme 16
F01.PB29801	3.81	30.73	1.89	17.50	36.56	1-Deoxy-D-Xylulose 5-Phosphate Synthase
F01.PB30289	3.81	20.48	0.38	94.05	20.48	3-Hydroxy-3-Methylglutaryl-Coenzyme A Reductase 3
F01.PB30570	65.21	35.09	51.61	23.55	19.36	Acyl-Activating Enzyme 5
F01.PB33429	21.55	14.18	2.69	4.60	10.45	Udp-Glycosyltransferase 87A1
F01.PB33635	5.85	18.84	28.70	12.15	12.21	Acyl-Activating Enzyme 1
F01.PB34557	0.08	0.52	0.00	13.43	34.53	Udp-Glucosyltransferase Family Protein
F01.PB3544	4.30	4.07	0.80	19.44	40.65	Cytochrome P450 71A1
F01.PB35649	1.79	7.21	20.64	16.28	20.17	Acetyl-Coenzyme A Carboxylase Carboxyl Transferase Subunit Alpha
F01.PB3977	46.53	28.49	4.19	0.21	10.64	Udp-Glycosyltransferase
F01.PB40488	5.50	6.18	3.77	7.33	14.63	Acetyl-Coenzyme A Carboxylase Carboxyl Transferase Subunit Alpha

F01.PB43	38.11	110.15	211.74	58.07	28.13	2-C-Methyl-D-Erythritol 2,4-Cyclodiphosphate Synthase
F01.PB432	63.66	38.25	146.61	20.94	199.77	Udp-Glucosyl Transferase 73B3
F01.PB46801	30.87	57.69	74.98	36.01	21.79	Chorismate Synthase 1
F01.PB46863	16.72	2.92	14.79	17.42	23.21	Udp-Glucosyl Transferase 85A2
F01.PB46950	17.22	14.91	2.92	44.48	145.41	Udp-Glycosyltransferase
F01.PB47063	62.46	23.56	2.41	13.73	12.49	Udp-Glucosyltransferase Family Protein
F01.PB47348	0.02	0.18	3.08	2.69	30.39	Hydroquinone Glucosyltransferase
F01.PB47552	0.02	0.08	0.06	0.13	12.03	Cytochrome P450 71A1
F01.PB47927	1.18	2.06	4.47	7.47	10.47	3-Dehydroquinate Synthase
F01.PB48318	4.00	4.27	6.42	9.42	12.42	Shikimate Dehydrogenase
F01.PB4860	0.49	13.86	94.92	13.44	19.45	Cytochrome P450 Monooxygenase Cyp89A28
F01.PB49420	56.97	42.64	24.42	18.26	22.85	Cyclin-Dependent Kinase
F01.PB49500	2.70	3.97	7.77	2.07	17.73	Protein PHYLLO, chloroplastic
F01.PB49521	12.34	10.73	0.00	34.07	41.40	Udp-Glycosyltransferase

F01.PB49848	5.08	33.61	6.04	11.15	22.08	1-Deoxy-D-Xylulose-5-Phosphate Synthase
F01.PB49988	21.15	18.34	15.06	15.29	15.67	Acyl-Activating Enzyme 17
F01.PB50624	0.06	0.75	0.03	3.80	14.16	3-Phosphoshikimate 1-Carboxyvinyltransferase
F01.PB5440	2.66	1.48	0.28	5.14	19.64	Udp-Glycosyltransferase
F01.PB5577	19.80	30.57	0.06	137.59	57.62	Udp-Glycosyltransferase
F01.PB5630	76.16	27.03	6.79	90.89	13.15	3-Ketoacyl-Coa Synthase 11
F01.PB5637	4.69	1.93	0.62	11.95	157.10	Udp-Glycosyltransferase
F01.PB6061	2.18	3.78	5.16	8.16	11.16	3-Dehydroquinate Synthase
F01.PB7847	36.20	89.05	190.84	353.99	20.71	Udp-Glycosyltransferase
F01.PB797	35.25	68.09	106.10	68.77	12.70	4-Hydroxy-3-Methylbut-2-Enyl Diphosphate Reductase
F01.PB7996	5.83	20.21	0.70	7.35	13.60	1-Deoxyxylulose-5-Phosphate Synthase
F01.PB8269	0.00	0.00	0.00	18.71	120.79	Udp-Glycosyltransferase
F01.PB8827	0.59	3.95	3.05	20.32	298.11	Udp-Glycosyltransferase
F01.PB8896	29.66	41.46	4.14	20.59	17.85	Phospho-2-Dehydro-3-Deoxyheptonate Aldolase 1

F01.PB9034	0.00	0.00	0.00	0.00	23.62	Udp-Glycosyltransferase
F01.PB9323	195.06	65.14	77.29	19.48	54.72	Udp-Glycosyltransferase
F01.PB9394	23.19	12.07	0.84	18.32	13.47	4-Hydroxy-3-Methylbut-2-Enyl Diphosphate Reductase
F01.PB9468	1.33	14.05	0.07	20.38	320.70	Cytochrome P450 71A26
F01.PB9810	0.17	0.11	2.13	8.10	205.83	Udp-Glycosyltransferase
F01.PB4662	81.60	86.47	81.13	186.78	161.05	Udp-Glycosyltransferase
F01.PB12588	18.64	21.86	17.19	14.18	17.26	Glabra2 Expression Modulator
F01.PB10	217.20	224.88	112.20	234.23	138.96	Late Embryogenesis Abundant Hydroxyproline-Rich Glycoprotein
F01.PB10002	19.20	14.78	9.66	7.16	14.69	Gata Transcription Factor 24
F01.PB10007	40.21	106.06	172.46	86.60	20.43	Myb Transcription Factor Myb118 Isoform X1
F01.PB10028	11.58	10.78	2.54	5.98	14.30	Zinc Finger Ccch Domain-Containing Protein 32-Like Isoform X1
F01.PB10228	43.47	132.59	1.74	44.27	12.48	Auxin-Responsive Protein Iaa27

F01.PB107	0.50	0.37	0.24	0.26	488.41	Transcription Factor Hbp-1B(C38)
F01.PB10770	0.42	2.43	32.41	0.43	16.56	Homeobox Protein, Putative
F01.PB10793	285.95	194.53	31.67	25.04	18.74	Homeobox-Leucine Zipper Protein Athb-6-Like Isoform X2
F01.PB11208	41.52	40.88	2.41	11.77	20.36	Bzip Transcription Factor Bzip124
F01.PB11229	62.38	69.00	63.82	40.18	28.95	Zinc Finger, C3Hc4 Type (Ring Finger) Family Protein Isoform 1
F01.PB11410	78.76	59.94	53.43	68.50	26.32	Transcription Factor Bhlh47
F01.PB11550	623.17	450.16	6.49	10.66	74.14	Nac Transcription Factor-Like Protein
F01.PB1180	0.00	0.00	0.00	0.00	11.76	Late Embryogenesis Abundant Protein D-34-Like
F01.PB11820	196.24	51.59	54.45	25.16	29.00	Zinc Finger Ccch Domain-Containing Protein 49
F01.PB11904	12.89	10.87	4.60	16.99	22.73	Protein Auxin Response 4
F01.PB1192	206.45	193.69	27.01	80.31	23.17	Auxin Response Factor 6-Like Isoform X3

F01.PB11937	174.75	246.80	29.77	98.68	53.30	Auxin-Responsive Protein Iaa9
F01.PB1207	44.37	27.70	27.83	26.86	28.06	Transcription Factor Bhlh14
F01.PB12154	0.04	0.64	0.00	15.10	104.94	Zinc Finger Ccch Domain-Containing Protein 2]
F01.PB12157	1.30	54.90	0.64	24.09	26.71	Transcription Factor Bhlh148
F01.PB12201	14.69	17.94	7.67	10.49	10.46	Zinc Finger Protein Magpie
F01.PB123	56.17	73.11	36.52	30.36	18.61	Transcription Factor Bhlh104
F01.PB12333	23.14	36.49	15.25	24.99	16.86	Nuclear Transcription Factor Y Subunit C-9
F01.PB12406	122.37	69.73	24.20	93.59	25.69	Putative Myb-Related Protein 20
F01.PB12499	105.11	104.44	215.65	25.98	21.60	Zinc Finger Protein Constans-Like 5
F01.PB12556	145.28	135.23	85.23	107.14	97.21	Transcription Factor Ilr3-Like Isoform 1
F01.PB12599	2.96	4.92	0.42	53.89	98.20	Zinc Finger Ccch Domain-Containing Protein 2-Like

F01.PB12611	0.08	0.05	1.04	74.22	14.55	K-Box Region And Mads-Box Transcription Factor Family Protein Isoform 1
F01.PB1262	161.32	122.68	23.67	63.60	70.53	Auxin-Responsive Protein Iaa9-Like Isoform X2
F01.PB12679	27.41	28.79	21.24	44.14	12.73	Ring Finger And Chy Zinc Finger Domain-Containing Protein 1-Like Isoform X1
F01.PB12856	96.10	62.76	12.39	28.51	21.43	Putative Wrky Transcription Factor 21
F01.PB13177	5.41	15.16	33.40	53.26	69.92	Zinc-Finger Homeodomain Protein 9
F01.PB13285	14.02	7.39	11.75	8.89	10.04	Transcription Factor Bhlh122-Like Isoform X1
F01.PB13301	0.78	0.59	0.14	8.58	14.52	Zinc Finger Ccch Domain-Containing Protein 2
F01.PB13756	3.03	3.12	2.53	0.00	13.72	Zinc Finger Ccch Domain-Containing Protein 11
F01.PB13831	38.00	52.00	40.70	28.48	49.71	Zinc Finger Ccch Domain-Containing Protein 40

F01.PB13938	89.38	16.88	16.57	23.29	14.09	Lob Domain-Containing Protein 38
F01.PB14074	318.80	237.25	60.93	118.86	19.31	Transcription Factor C2H2
F01.PB14103	7.22	14.96	23.96	33.29	11.03	Late Embryogenesis Abundant Hydroxyproline-Rich Glycoprotein
F01.PB14123	11.12	7.99	12.04	77.05	13.14	Nac-Like Transcription Factor
F01.PB14289	88.92	172.24	50.50	44.10	16.08	Homeobox Protein, Putative Isoform 1
F01.PB14325	20.78	20.62	25.79	18.08	10.91	Bzip Transcription Factor Bzip56 Precursor
F01.PB14399	18.00	18.92	7.33	41.59	63.63	Myb Family Transcription Factor
F01.PB14532	48.69	53.77	32.41	41.73	41.90	Nuclear Transcription Factor Y Subunit C-9
F01.PB14683	0.95	0.21	0.32	1.15	20.49	Transcription Factor Bhlh67
F01.PB14812	30.94	11.41	4.79	5.97	13.11	Morc Family Cw-Type Zinc Finger Protein 4
F01.PB1490	125.22	72.81	14.98	100.85	17.22	Putative Myb-Related Protein 20
F01.PB15014	144.86	55.78	33.31	55.86	179.77	Nac Domain-Containing

						Protein 100
F01.PB15050	27.61	26.51	18.78	21.09	12.13	Atp-Dependent Zinc Metalloprotease Ftsh 4, Mitochondrial
F01.PB15377	0.00	0.06	0.00	0.00	37.11	Predicted: Mads-Box Transcription Factor 18-Like Isoform X1
F01.PB1543	33.77	23.37	12.85	12.02	17.01	Zinc Finger Ccch Domain-Containing Protein 40
F01.PB15433	16.66	19.14	10.97	8.88	15.52	Squamosa Promoter-Binding-Like Protein
F01.PB15651	5.30	23.26	12.00	11.25	21.20	Zinc Finger Protein Jackdaw
F01.PB15751	69.36	91.76	20.49	60.46	81.92	Auxin-Responsive Protein Iaa9-Like Isoform X1
F01.PB15956	144.47	70.52	19.86	24.02	24.46	Gras Family Transcription Factor
F01.PB16052	56.10	23.32	13.77	12.14	26.31	Zinc Finger Ccch Domain-Containing Protein 25-Like Isoform X1
F01.PB16108	81.48	43.82	21.92	35.10	12.12	Wrky Transcription Factor 17-Like

F01.PB16131	0.15	0.08	0.08	16.21	108.74	Zinc Finger Family Protein
F01.PB16143	188.81	140.79	101.24	67.45	10.88	Zinc Finger A20 And An1 Domain-Containing Stress-Associated Protein 5
F01.PB16222	13.27	9.45	0.70	65.71	41.05	Ring Finger And Chy Zinc Finger Domain-Containing Protein 1
F01.PB16242	110.42	86.96	29.70	26.28	14.85	Homeobox Protein Knotted-1-Like 7
F01.PB16338	397.62	202.35	29.02	78.44	52.02	Nac Domain Protein
F01.PB16499	43.94	27.57	31.04	16.79	88.73	Nuclear Transcription Factor Y Subunit A-1
F01.PB16529	50.03	33.82	24.47	24.81	24.23	Zinc Finger Protein 207
F01.PB16736	10.07	9.58	9.86	7.46	14.26	C2H2 Type Zf-Met: Zinc-Finger Protein
F01.PB16736	10.07	9.58	9.86	7.46	14.26	C2H2 Type Zf-Met: Zinc-Finger Protein
F01.PB1685	163.23	131.53	12.60	36.10	79.04	Chy And Ctchy And Ring-Type Zinc Finger Proteintruncatula]
F01.PB16852	37.09	21.39	12.62	16.78	10.27	Gamyb-Binding Protein Family Protein

F01.PB16996	0.00	0.07	0.07	4.19	20.82	Protein Yabby 4
F01.PB17056	34.37	38.45	15.80	19.28	21.49	Transcription Factor Bhlh148-Like
F01.PB17111	45.18	55.64	32.02	26.52	11.15	Transcription Factor C2H2
F01.PB17249	32.94	34.25	29.59	17.61	14.21	Zinc Finger Protein
F01.PB17278	42.28	22.80	19.11	7.25	29.21	Nuclear Transcription Factor Y Subunit A-7-Like Isoformx2
F01.PB17297	50.62	36.28	48.02	318.54	47.22	Nac Transcription Factor-Like Protein
F01.PB17408	51.25	31.66	27.76	19.12	37.08	Rna-Binding Family Protein With Retrovirus Zinc Finger-Like Domain, Putative
F01.PB18278	83.43	67.07	2.72	14.66	30.90	Bzip Transcription Factor Bzip124
F01.PB1833	38.69	38.84	30.53	46.83	23.70	Nac Domain-Containing Protein 78-Like Isoform X2
F01.PB18393	134.53	192.00	86.99	36.92	38.97	Homeobox-Leucine Zipper Protein Hat5
F01.PB18559	0.10	7.55	128.57	7.86	21.04	Gata Transcription Factor 21
F01.PB18592	0.31	0.17	0.18	0.23	675.10	Late Embryogenesis

						Abundant Protein D-34
F01.PB18668	4.58	1.98	6.41	5.78	13.80	Nuclear Transcription Factor Y Subunit A-7
F01.PB18696	0.00	0.00	1.50	0.00	14.14	Nac Domain-Containing Protein 2-Like
F01.PB187	1708.02	930.11	765.44	4164.14	80.79	Putative Late Embryogenesis Abundant Protein
F01.PB18702	0.58	4.83	0.00	3.37	13.21	Trihelix Transcription Factor Gtl2-Like Isoform X1
F01.PB18724	8.56	9.80	8.79	0.55	24.81	Zinc-Finger Homeodomain Protein 9-Like
F01.PB18751	1.88	4.99	3.75	6.18	11.18	Zinc Finger Protein Zat4
F01.PB18914	5.78	6.63	10.70	5.52	10.88	Homeobox Protein Luminidependens
F01.PB19012	0.18	2.00	1.19	104.61	295.28	Mads Box Protein M8
F01.PB19296	0.06	0.35	0.14	4.86	17.65	Protein Yabby 4
F01.PB19338	32.24	38.03	27.52	27.79	17.43	Zinc Transporter Ztp29 Isoform X1
F01.PB19415	6.21	36.81	23.68	33.52	14.04	Myb-Related Transcription Factor
F01.PB19453	326.51	331.89	80.68	13.10	17.51	Homeobox Protein,

						Putative Isoform 2
F01.PB19594	10.02	15.02	21.94	2.93	11.11	B-Box Type Zinc Finger Family Protein
F01.PB19638	23.95	27.77	21.14	22.17	19.25	Alcohol Dehydrogenase Transcription Factor Myb/Sant-Like Family Protein
F01.PB19690	51.53	36.22	5.62	12.96	31.92	Homeobox-Leucine Zipper Protein Revoluta
F01.PB1975	80.94	41.38	12.20	12.10	10.80	Probable Wrky Transcription Factor 32 Isoform X1
F01.PB19780	19.67	14.52	10.00	10.88	12.25	Zinc Finger Ccch Domain-Containing Protein 13
F01.PB20184	35.70	22.13	4.48	34.40	10.03	Zinc Finger Protein Constans-Like Protein
F01.PB2029	57.55	57.35	18.07	14.94	23.39	Protein Auxin Signaling F-Box 2-Like, Partial
F01.PB20390	146.00	93.00	38.92	23.29	58.58	Homeobox-Leucine Zipper Protein 4 / Hd-Zip Protein
F01.PB20463	17.66	42.18	51.21	25.42	28.93	Zinc Finger Protein Constans-Like 13-Like
F01.PB20618	91.17	84.81	35.72	18.88	17.63	Homeobox-Leucine

					Zipper Protein Hat5
F01.PB20902	10.53	10.82	6.46	4.05	11.61	Gata Transcription Factor 24
F01.PB21142	20.33	17.38	3.61	10.56	62.74	Transcription Factor Bhlh93
F01.PB21330	62.70	85.14	25.99	14.87	20.26	Transcription Factor Lhw
F01.PB21339	109.79	145.21	43.80	77.54	58.22	Nuclear Transcription Factor Y Subunit C-9
F01.PB21351	70.42	58.27	21.96	16.66	23.44	Wrky Transcription Factor 32
F01.PB21364	15.69	4.98	0.57	4.86	18.69	Auxin Response Factor 5
F01.PB21549	330.10	291.35	11.89	152.82	41.40	Auxin-Responsive Protein Iaa16-Like
F01.PB21596	122.07	75.72	42.49	125.74	11.55	Transcription Factor Bhlh14
F01.PB21671	35.28	27.41	19.10	17.44	12.19	Zinc Finger Ccch Domain-Containing Protein 17
F01.PB21869	128.24	97.97	45.81	24.94	18.86	Wrky Protein Isoform 1
F01.PB21888	51.54	42.36	26.17	34.07	26.74	Tcp-1/Cpn60 Chaperonin Family Protein Isoform 1
F01.PB22021	33.62	16.85	5.93	24.24	12.56	Gata Transcription Factor 8-Like Isoform X2
F01.PB22694	19.80	27.93	2.29	14.75	13.45	Dof Zinc Finger Protein

						Dof2.1-Like
F01.PB22777	0.11	0.03	0.00	6.04	24.48	K-Box Region And Mads-Box Transcription Factor Family Protein Isoform 1
F01.PB22985	28.13	50.89	100.25	126.61	14.88	Zinc Finger Ccch Domain-Containing Protein Zfn-Like Isoformx2
F01.PB23062	761.96	380.30	104.36	215.56	26.19	Nac Domain Protein
F01.PB23090	199.40	131.53	78.54	107.93	17.38	Transcription Factor C2H2
F01.PB23106	36.45	25.12	33.36	32.06	24.35	Zinc Finger Protein 622
F01.PB2315	118.27	100.61	60.21	98.30	42.22	Squamosa Promoter-Binding-Like Protein 12
F01.PB23201	641.64	238.87	115.06	229.13	21.23	Zinc Finger Protein
F01.PB2321	38.71	39.77	32.07	24.79	14.46	Nuclear Transcription Factor Y Subunit C-1
F01.PB23270	38.80	20.73	27.65	16.14	21.47	Zinc Finger Ccch Domain-Containing Protein 1
F01.PB23372	17.56	37.73	60.92	84.08	73.82	Late Embryogenesis Abundant Protein Lea14-A, Putative

F01.PB2363	86.35	37.26	28.86	26.92	35.96	Bzip Transcription Factor Bzip78
F01.PB23633	11.96	29.04	43.58	14.85	15.44	Auxin Response Factor
F01.PB23645	58.70	37.93	20.52	24.88	16.25	Gras Family Transcription Factor
F01.PB23665	67.46	62.14	52.91	47.98	15.25	Transcription Factor Bhlh143
F01.PB23723	13.36	21.01	37.39	39.25	29.89	Atp-Dependent Zinc Metalloprotease Ftsh 10, Mitochondrial
F01.PB23739	16.12	19.77	25.36	19.99	11.72	Nac Domain-Containing Protein 78
F01.PB23919	15.43	15.29	22.18	39.74	30.11	Tcp-1/Cpn60 Chaperonin Family Protein
F01.PB2405	350.74	121.41	125.47	105.58	52.66	Nac Domain-Containing Protein 2
F01.PB24072	34.54	1.00	0.33	20.59	220.58	Auxin-Responsive Gh3 Family Protein
F01.PB24083	35.67	29.94	20.88	19.78	17.86	Zinc Finger Cchc Domain-Containing Protein 7
F01.PB2410	183.60	33.59	24.46	46.58	29.92	Lob Domain-Containing Protein 38
F01.PB24154	8.36	24.69	177.73	48.32	12.39	Atp-Dependent Zinc

						Metalloprotease Ftsh 2
F01.PB24253	15.95	16.33	12.80	37.87	24.87	Zinc Finger C-X8-C-X5-C-X3-H Type Family Protein
F01.PB24376	34.95	31.46	19.96	23.19	29.11	Transcription Factor Bhlh3
F01.PB24488	6.57	6.49	3.68	11.77	14.74	Zinc Finger Ccch Domain-Containing Protein
F01.PB24648	18.68	32.85	32.95	28.02	28.12	Nac Transcription Factor 044
F01.PB24858	1.63	9.63	26.15	2.18	10.94	Zinc Finger Protein Magpie
F01.PB25741	0.97	4.09	16.80	1.67	15.25	Zinc Finger Protein Magpie-Like Isoform X1
F01.PB25934	141.52	21.05	14.45	128.65	13.82	Ring Finger And Chy Zinc Finger Domain-Containing Protein 1
F01.PB27486	27.49	46.66	20.31	11.42	14.03	Zinc Finger Protein Constans-Like 9
F01.PB27782	26.46	25.72	21.09	16.56	15.27	Zinc Finger Ccch Domain-Containing Protein 13
F01.PB2811	143.07	118.02	34.64	31.14	26.55	Homeobox-Leucine

						Zipper Protein Hat5
F01.PB28873	43.75	20.90	5.54	35.52	35.47	Homeobox-Leucine Zipper Protein Glabra 2
F01.PB28873	43.75	20.90	3.78	35.52	35.47	Homeobox-Leucine Zipper Protein Glabra 2, Partial
F01.PB2934	11.60	19.76	12.85	11.83	14.00	Transcription Factor C2H2
F01.PB2975	57.82	48.30	28.43	32.37	23.91	Transcription Factor Hbp-1A
F01.PB29862	26.66	31.53	62.70	54.71	36.74	Nac Domain-Containing Protein 78-Like Isoform X1
F01.PB30023	23.23	14.75	14.83	16.96	16.85	C2H2-Like Zinc Finger Protein
F01.PB30023	23.23	14.75	14.83	16.96	16.85	C2H2-Like Zinc Finger Protein
F01.PB30072	28.20	16.24	5.42	18.00	26.34	Zinc Finger Protein Nutcracker
F01.PB30173	16.58	11.10	0.98	4.75	11.34	Homeobox-Leucine Zipper Protein Revoluta
F01.PB30194	3.52	19.03	23.20	17.46	16.11	Transcription Factor Bhlh3-Like Isoform X1
F01.PB303	64.50	115.95	0.61	18.10	13.44	Auxin-Responsive Protein Iaa27

F01.PB30557	22.81	13.00	15.22	18.95	18.41	Zinc Finger Ccch Domain-Containing Protein
F01.PB30997	13.24	10.06	7.95	7.33	14.62	Myb-Like Protein X-Like Isoform X1
F01.PB31220	18.29	13.71	10.45	10.88	13.31	Ankyrin Repeat And Zinc Finger Domain-Containing Protein 1-Like Isoform X2
F01.PB31450	18.34	9.79	12.08	9.64	31.39	Nuclear Transcription Factor Y Subunit A-1
F01.PB31483	22.35	8.02	6.18	35.67	22.94	Ring Finger And Chy Zinc Finger Domain-Containing Protein 1
F01.PB31676	9.31	11.99	8.82	10.27	12.88	Zinc Finger Ccch Domain-Containing Protein 53-Like Isoform X1
F01.PB3189	32.53	31.10	14.05	24.77	25.57	Late Embryogenesis Abundant Protein Lea14-A
F01.PB32560	60.01	25.27	10.18	12.11	14.00	Low Quality Protein: Transcription Factor Tcp8
F01.PB33011	13.44	8.82	1.48	9.83	14.15	Predicted: Auxin Response Factor 9

F01.PB33304	13.78	15.55	49.67	27.46	72.58	Trihelix Transcription Factor Gt-2
F01.PB33839	1.75	17.64	7.03	14.60	15.52	Trihelix Transcription Factor Gtl2-Like Isoform X1
F01.PB34396	44.43	40.20	8.40	22.66	16.40	Putative Wrky Transcription Factor 21
F01.PB34408	3.88	10.39	123.53	43.47	12.00	Atp-Dependent Zinc Metalloprotease Ftsh 2
F01.PB35492	2.54	17.63	100.49	24.03	14.58	Atp-Dependent Zinc Metalloprotease Ftsh 2
F01.PB35614	14.38	19.51	24.72	17.16	15.02	Zinc-Metallopeptidase, Peroxisomal-Like Isoform X2
F01.PB35640	16.47	10.95	8.88	14.08	21.66	Ring Finger And Chy Zinc Finger Domain-Containing Protein 1
F01.PB35768	29.49	15.06	16.53	23.40	16.46	Atp-Dependent Zinc Metalloprotease Ftsh 10
F01.PB3605	4.57	5.54	0.78	2.81	10.17	Gata Transcription Factor 16
F01.PB36117	39.73	28.09	15.06	17.15	19.10	Zinc Finger Protein 598
F01.PB36157	12.87	20.22	23.94	19.57	13.82	Squamosa Promoter-Binding-Like

						Protein 12
F01.PB36406	15.05	12.52	3.55	12.50	15.75	Homeobox-Leucine Zipper Protein Revoluta
F01.PB36694	28.11	21.44	2.40	14.54	18.08	Homeobox-Leucine Zipper Protein Revoluta
F01.PB37055	12.83	10.50	7.65	9.61	11.20	Bromodomain Adjacent To Zinc Finger Domain Protein 1A
F01.PB3780	174.17	343.68	396.55	270.25	24.59	Myb Transcription Factor Myb156
F01.PB3829	0.00	0.00	0.00	0.02	22.20	Myb-Related Protein Myb4
F01.PB38904	18.71	18.30	4.58	93.08	38.41	Ring Finger And Chy Zinc Finger Domain-Containing Protein 1
F01.PB39154	10.50	26.63	18.09	27.36	14.79	Squamosa Promoter-Binding-Like Protein 12
F01.PB3944	26.00	39.60	33.73	29.76	21.35	Transcription Factor Bhlh113-Like Isoform X2
F01.PB3952	34.61	43.78	15.10	12.80	10.02	14 Kda Zinc-Binding Protein
F01.PB40141	2.46	2.34	4.53	9.98	10.21	Atp-Dependent Zinc Metalloprotease Ftsh 2

F01.PB4026	790.68	204.97	87.88	58.54	10.56	Putative Zinc-Binding Protein
F01.PB40675	64.13	28.56	19.19	23.80	35.10	Auxin Response Factor 1-Like Isoform X1
F01.PB409	0.17	0.18	0.12	13.20	157.06	Agamous-Like Mads-Box Protein Agl11 Isoform X3
F01.PB41445	16.91	13.16	9.65	6.51	12.94	Zinc Ion-Binding Protein
F01.PB41678	7.18	11.00	12.30	9.98	21.72	Zinc Finger Ccch Domain-Containing Protein 53-Like Isoform X1
F01.PB42383	3.99	3.21	0.63	3.21	15.46	Wrky Transcription Factor 32
F01.PB42544	19.80	17.36	5.73	11.13	11.73	Homeobox Protein Luminidependens
F01.PB42615	24.51	33.57	16.57	7.95	12.79	Auxin Response Facto
F01.PB43929	34.64	58.44	42.43	39.84	31.43	Zinc Finger Ccch Domain-Containing Protein 30
F01.PB44833	146.77	50.13	1.75	18.36	19.31	Putative Cadmium/Zinc-Transporting Atpase Hma4
F01.PB46103	18.45	30.80	48.65	32.26	17.52	Auxin Response Factor
F01.PB46224	9.54	9.03	19.70	14.11	10.57	Squamosa Promoter-Binding-Like

						Protein 12-Like
F01.PB46433	19.18	25.67	12.82	6.03	31.53	Zinc/Ring Finger Protein
F01.PB46565	0.40	0.64	1.41	19.30	890.58	Late Embryogenesis Abundant Protein Group 3, Putative
F01.PB46590	41.70	94.76	13.20	74.40	18.51	Ring-H2 Zinc Finger Protein Rha1A
F01.PB46762	78.48	108.54	12.95	55.33	17.67	Auxin-Responsive Protein Iaa9
F01.PB46839	11.63	18.84	25.27	16.52	22.68	Myb-Like Protein X-Like Isoform X6
F01.PB46867	134.40	90.92	14.91	7.11	12.15	Homeobox-Leucine Zipper Protein Hat22
F01.PB46923	12.60	33.91	36.83	18.67	24.95	Zinc Transporter 6, Chloroplastic
F01.PB47101	3.25	9.73	2.53	7.72	14.17	Gata Transcription Factor
F01.PB4739	325.45	217.24	316.31	269.01	96.73	Nac Domain-Containing Protein 2
F01.PB47406	5.35	18.88	0.84	2.99	12.23	Transcription Factor Myb86
F01.PB4743	695.97	350.20	48.57	169.29	17.17	Auxin-Responsive Protein Iaa16
F01.PB47777	32.76	30.30	16.74	20.07	11.72	Wrky Transcription Factor

						15 Family Protein
F01.PB47802	45.21	41.70	3.18	13.57	18.56	Dof Zinc Finger Protein Dof2.1
F01.PB47802	45.21	41.70	3.18	13.57	18.56	Dof Zinc Finger Protein Dof2.1
F01.PB48100	0.04	2.16	0.00	0.27	13.76	Transcription Factor Myb86
F01.PB48118	34.84	22.42	21.26	15.88	19.85	Zinc Finger Ccch Domain-Containing Protein 48
F01.PB48170	50.83	40.50	38.02	72.43	44.78	Atp-Dependent Zinc Metalloprotease Ftsh 10
F01.PB4830	263.42	401.09	168.21	89.48	59.11	Homeobox Protein, Putative Isoform 1
F01.PB48499	54.51	47.35	20.67	16.78	21.85	Auxin Signaling F-Box Protein 2
F01.PB48544	0.66	1.10	0.65	12.11	16.69	Transcription Factor Tcp4
F01.PB4856	106.85	104.01	49.01	50.61	63.21	Zinc Finger Ccch Domain-Containing Protein 43
F01.PB48668	37.13	32.53	25.71	40.48	20.92	Nac Domain-Containing Protein 78
F01.PB48794	125.24	36.05	12.89	25.44	14.57	Homeobox-Leucine Zipper Protein Athb

F01.PB48807	9.30	17.65	13.88	12.58	18.64	Zinc Finger Ccch Domain-Containing Protein 56
F01.PB48908	0.22	0.13	0.08	0.74	465.30	Late Embryogenesis Abundant Protein D-34
F01.PB48947	25.13	17.85	1.02	2.43	15.77	Nac Domain-Containing Protein 8
F01.PB49035	14.23	16.62	2.91	9.88	11.66	Gata Transcription Factor 5
F01.PB49074	0.00	0.02	54.20	0.32	11.72	Putative Gata Transcription Factor 22
F01.PB49173	0.09	1.15	0.13	47.70	34.68	Zinc Finger Homeodomain Protein Szf-Hd1
F01.PB49352	105.48	152.55	96.74	81.10	37.32	Transcription Factor Bhlh144
F01.PB49546	89.88	102.01	94.08	29.84	16.69	Nuclear Transcription Factor Y Subunit B-2
F01.PB50180	193.66	113.66	53.70	23.35	22.71	Gata Transcription Factor 24-Like Isoformx1
F01.PB5076	32.47	211.72	271.38	66.33	22.80	B-Box Zinc Finger Protein 32
F01.PB5085	38.23	16.62	18.77	17.35	63.26	Zinc Finger C-X8-C-X5-C-X3-H Type Family Protein, Putative

F01.PB51016	15.18	15.31	20.21	20.08	19.69	Transcription Factor Bhlh49-Like Isoform X2
F01.PB51097	14.76	12.88	8.16	8.72	11.26	Zinc Finger Ccch Domain-Containing Protein 64
F01.PB5125	340.99	296.27	142.28	75.26	91.15	Trihelix Transcription Factor Asil1
F01.PB52031	9.78	4.96	9.25	14.59	18.42	Atp-Dependent Zinc Metalloprotease Ftsh 2
F01.PB52040	39.84	51.28	26.97	24.59	16.10	Transcription Factor Bhlh143
F01.PB5266	24.37	13.25	3.45	9.60	179.88	Nuclear Transcription Factor Y Subunit A-9
F01.PB54457	2.53	3.44	8.32	4.55	11.22	Trihelix Transcription Factor Gt-2
F01.PB54738	10.11	6.25	5.39	27.27	11.75	Ring Finger And Chy Zinc Finger Domain-Containing Protein 1
F01.PB5579	58.42	34.93	46.79	22.56	15.09	Myb Domain Protein 3, Putative
F01.PB55961	12.24	11.19	8.27	6.81	12.84	Zinc Finger Ccch Domain-Containing Protein 69
F01.PB56112	20.60	11.79	8.11	9.07	13.30	Myb Transcription Factor

F01.PB5631	0.00	5.05	2.77	0.00	11.04	Nac Domain-Containing Protein 2
F01.PB5633	59.43	54.24	45.97	30.77	16.84	Nuclear Transcription Factor Y Subunit A-7
F01.PB5638	186.62	57.74	39.17	30.24	21.08	Gata Transcription Factor 5-Like Isoform X1
F01.PB5671	302.52	244.13	163.43	112.43	87.47	Transcription Factor Myb1R1
F01.PB5683	55.66	54.86	56.15	20.46	39.66	Putative Myb-Related Protein 23
F01.PB5689	101.54	83.49	99.17	23.25	15.18	Nac Domain-Containing Protein 2
F01.PB5702	34.20	23.86	10.24	7.03	12.21	Putative R2R3 Myb Protein 5
F01.PB57075	3.73	5.39	8.58	10.46	17.19	Atp-Dependent Zinc Metalloprotease Ftsh 2
F01.PB5808	194.20	245.74	14.97	82.31	143.61	Bzip Transcription Factor Bzip124
F01.PB5815	146.82	330.39	60.35	121.63	40.62	Bzip Transcription Factor Bzip124
F01.PB5974	0.00	0.00	0.00	0.00	19.69	Mads-Box Transcription Factor 18
F01.PB5984	10.48	10.67	27.31	16.86	26.91	Predicted: Transcription Factor Myb44

F01.PB6021	59.10	27.39	18.86	12.90	10.61	Auxin Response Factor
F01.PB6125	18.85	16.02	16.56	20.03	16.33	Zinc Finger Protein Zpr1 Homolog Isoformx1
F01.PB6309	36.38	28.91	10.70	8.09	12.38	Zinc Finger Protein Constans-Like 9
F01.PB6426	47.81	34.83	19.63	34.89	19.53	Predicted: Transcription Factor Tcp7
F01.PB6443	49.95	27.12	24.83	22.27	24.07	Zinc Finger Protein 830
F01.PB652	230.14	64.07	33.26	66.74	29.83	Lob Domain-Containing Protein 37
F01.PB6521	17.58	40.32	240.36	142.07	17.71	Atp-Dependent Zinc Metalloprotease Ftsh 2
F01.PB6572	51.75	37.48	34.58	40.83	27.28	Zinc Finger Ccch Domain-Containing Protein 11
F01.PB6578	0.05	1.40	0.00	0.52	16.38	Transcription Factor Myb86-Like
F01.PB6621	4.06	6.75	1.47	11.62	28.54	Gata Transcription Factor 5-Like
F01.PB6711	56.37	121.23	108.18	115.94	12.95	Myb Transcription Factor Myb62 Isoform X1
F01.PB6716	41.16	21.79	3.76	16.49	23.02	Zinc Finger Protein Nutcracker
F01.PB6777	29.38	18.34	28.15	19.07	25.62	Zinc Finger Protein

						Gis2-Like Isoform X1
F01.PB6853	38.97	31.46	34.00	21.49	26.16	Nac Domain-Containing Protein 78
F01.PB7074	7.53	16.28	26.70	10.60	64.95	Nuclear Transcription Factor Y Subunit A-1
F01.PB7319	110.43	23.58	28.82	11.64	11.04	Zinc Finger Ccch Domain-Containing Protein 49
F01.PB755	101.68	143.80	7.33	11.51	12.58	Auxin-Responsive Protein Iaa27
F01.PB7641	20.32	51.17	82.64	16.09	24.92	Nuclear Transcription Factor Y Subunit C-3
F01.PB7772	19.26	13.73	9.70	18.40	14.45	Transcription Factor Bhlh49-Like Isoform X2
F01.PB7782	189.33	35.33	16.28	158.78	29.30	Ring Finger And Chy Zinc Finger Domain-Containing Protein 1-Like Isoform X1
F01.PB7839	1.72	7.79	0.00	16.93	37.36	Agamous-Like Mads-Box Protein Agl11
F01.PB7863	61.72	68.07	19.48	33.84	18.93	Myb Family Transcription Factor Apl-Like Isoform X4

F01.PB798	125.70	116.16	84.71	118.10	40.89	Zinc Finger A20 And An1 Domain-Containing Stress-Associated Protein 1
F01.PB80	558.98	402.61	230.82	259.68	56.27	Zinc Finger A20 And An1 Domain-Containing Stress-Associated Protein 5
F01.PB8040	73.74	86.54	81.15	252.89	16.57	Nac Domain-Containing Protein 72
F01.PB8196	35.88	33.21	17.76	22.03	10.36	Squamosa Promoter-Binding-Like Protein 12
F01.PB8218	58.25	12.28	5.82	3.18	10.91	Gata Transcription Factor 24
F01.PB8265	10.10	48.33	17.75	7.91	13.97	Transcription Factor Myb1R1
F01.PB8316	16.19	7.81	31.12	7.79	34.35	Auxin Response Factor
F01.PB8332	10.96	32.66	5.08	13.44	16.76	Auxin-Responsive Protein Iaa9
F01.PB835	20.32	24.55	21.84	49.40	45.86	Nuclear Transcription Factor Y Subunit B-8, Partial
F01.PB8399	145.89	109.46	23.57	14.34	17.79	Transcription Factor Bhlh145

F01.PB8473	33.18	28.96	32.57	15.96	30.42	Predicted: Zinc Finger Ccch Domain-Containing Protein 20
F01.PB8557	0.00	0.00	0.10	86.15	21.45	Mads-Box Transcription Factor 12
F01.PB8670	22.20	19.59	9.82	15.52	12.61	Zinc-Metallopeptidase, Peroxisomal-Like Isoform
F01.PB8684	75.02	69.06	56.73	53.45	28.70	Glabra2 Expression Modulator
F01.PB8701	18.40	26.53	23.78	15.48	26.88	Trihelix Transcription Factor Gt-1
F01.PB8823	1.49	3.66	20.17	7.28	56.04	Zinc Finger Protein Magpie-Like Isoform X1
F01.PB8965	44.98	30.78	15.15	12.99	18.75	Gamyb-Binding Protein Family Protein
F01.PB8983	84.97	56.49	25.09	50.05	23.55	Transcription Factor Tcp7-Like
F01.PB9224	0.04	0.19	2.47	85.70	19.73	K-Box Region And Mads-Box Transcription Factor Family Protein Isoform 1
F01.PB9253	43.93	34.75	11.62	128.96	58.61	Ring Finger And Chy Zinc Finger Domain-Containing Protein 1

F01.PB9260	27.02	27.86	30.36	96.20	35.87	Bzip Transcription Factor Bzip68
F01.PB9276	12.64	13.55	6.42	14.04	15.98	Zinc Finger Protein-Like 1
F01.PB9305	29.43	23.54	16.16	13.34	10.11	Zinc Finger Ccch Domain-Containing Protein 69
F01.PB9343	128.62	50.04	1.31	9.39	12.87	Auxin-Responsive Protein Iaa13, Putative
F01.PB9552	60.58	43.75	33.98	29.75	20.04	Zinc Finger, C3Hc4 Type (Ring Finger) Protein
F01.PB9572	76.52	46.14	30.16	23.22	27.91	Transcription Factor Bhlh148-Like
F01.PB9609	44.31	38.98	32.68	27.53	19.19	Zinc Finger Protein Gis2-Like Isoform X1
F01.PB9796	69.67	46.72	94.91	13.74	35.17	Nuclear Transcription Factor Y Subunit A-3-Like Isoform X1

Table S3. Putative genes involved in anthraquinone biosynthesis identified in the root, stem, leaf, flower and seed of *C. obtusifolia*.

Pathway	Gene	Symbol	Number in root	Number in stem	Number in leaf	Number in flower	Number in seed
Mevalonate pathway	Acetyl-CoA C-acetyltansferase	ACAT	20	19	23	23	19
	Hydroxymethylglutaryl-CoA synthase	HMGS	3	3	3	3	3
	Hydroxymethylglutaryl-CoA reductase	HMGR	9	10	9	13	10
	Mevalonate kinase	MK	3	3	3	3	3
	Phosphomevalonate kinase	PMK	3	3	3	3	3
	Diphosphomevalonate decarboxylase	MPD	2	2	2	2	2
MEP pathway	1-deoxy-D-xylulose-5-phosphate synthase	DXS	9	9	9	9	9
	1-deoxy-D-xylulose-5-phosphate reductoisomerase	DXR	7	11	18	14	6
	2-C-methyl-D-erythritol 4-phosphate cytidylyltransferase	ISPD	2	3	2	2	3
	4-diphosphocytidyl-2-C-methyl-D-erythritol kinase	CDPMEK	4	4	4	4	3
	2-C-methyl-D-erythritol 2，4-cyclodiphosphate Synthase	ISPF	1	1	1	1	1
	（E)-4-hydroxy-3-methylbut-2-enyl-diphos	HDS	3	3	3	3	3

	phase synthase						
	4-hydroxy-3-methylbut-2-enyl-diphosphase reductase	HDR	11	10	10	9	10
	Isopentenyl-diphosphate delta-isomerase	IPP	11	10	9	12	11
Shikimate pathway	3-deoxy-7-phosphoheptulonate synthase	DAHPS	8	8	8	8	8
	3-dehydroquinate synthase	DHQS	4	4	4	4	4
	3-dehydroquinate dehydratase/ shikimate dehydrogenase	SDH/DHQD	1	1	1	2	1
	Shikimate kinase	SMK	6	6	7	6	6
	3-phosphoshikimate 1-carboxyvinyltransferase	EPSP synthase	7	7	7	7	7
	Chorismate synthase	CS	8	8	6	6	7
Menaqunone pathway	Menaquinone-specific isochorismate synthase	ICS	2	2	2	2	2
	Isochorismate synthase	ICS					
	2-succiny-6-hydroxy-2,4-cyclohexadiene-1-carboxylate synthase/O-succinyibenzoate synthase	menC/PHYLLO	3	3	2	2	3
	2-succinyl-6-hydroxy-2,4-cyclohexadiene-1-carboxylate synthase	MenH					
	2-succinyl-5-enolpyru	menD					

	vyl-6-hydroxy-3-cyclohexene-1-carboxylate synthase						
	Acyl-activating enzyme	menE	40	40	40	41	37
	Naphthoate synthase	menB	2	2	2	2	2
Polyketide pathway	Polyketide synthaseIII	PSKIII	9	10	10	10	9
	Polyketide cyclase/dehydratase	PKC	3	3	3	3	3
Glycosylation	UDP-Glucosyl transferase	UDPG	90	96	96	99	82
CYPs	Cytochrome p450	CYP	112	126	120	127	109
	Cytochrome p450 Monooxygenase	—	4	5	5	6	5
	NADPH-cytochrome p450 reductase	—	19	20	19	19	21

Table S4. List of CYPs of different plant species used for phylogenetic analysis

CYP name	Species	Accesssion id	Length
NADPH-cytochrome p450 reductase	Arachis duranensis	XP_015934960.1	692
CYP714A1	Arachis duranensis	XP_015946736.1	533
CYP71A1	Arachis duranensis	XP_015954538.1	522
CYP716B1	Arachis duranensis	XP_015956144.1	473
CYP85A	Arachis duranensis	XP_015962617.1	468
CYP71A26	Arachis duranensis	XP_015964672.1	505
CYP83B1	Arachis duranensis	XP_015969861.1	514
CYP98A2	Arachis duranensis	XP_015970644.1	508
NADPH-cytochrome p450 reductase	Arachis ipaensis	XP_016163722.1	692
CYP71A1	Arachis ipaensis	XP_016187643.1	527

CYP71A26	Arachis ipaensis	XP_016189615.1	505
CYP85A	Cajanus cajan	KYP49608.1	466
CYP716B2	Cajanus cajan	KYP76833.1	468
NADPH-cytochrome p450 reductase	Cajanus cajan	XP_020211814.1	691
CYP71A1	Cajanus cajan	XP_020216626.1	522
CYP714A1	Cajanus cajan	XP_020229038.1	532
CYP97B2	Cajanus cajan	XP_020230175.1	580
CYP98A2	Cicer arietinum	XP_004493325.1	527
CYP734A1	Cicer arietinum	XP_004495852.1	518
CYP71A1	Cicer arietinum	XP_004499226.1	513
CYP714A1	Cicer arietinum	XP_004502797.1	530
CYP71A26	Cicer arietinum	XP_004505028.1	500
CYP71A26	Cicer arietinum	XP_004515856.1	504
CYP97B1	Cicer arietinum	XP_012574398.1	580
CYP51	Corchorus olitorius	OMO51606.1	486
CYP709B2	Durio zibethinus	XP_022769769.1	523
CYP89H3	Glycine max	ABC68408.1	373
CYP51G1	Glycine max	ABC68412.1	475
CYP97B2	Glycine max	NP_001235534.2	576
CYP85A	Glycine max	NP_001241912.2	464
NADPH-cytochrome p450 reductase	Glycine max	XP_003526551.1	691
CYP714A1	Glycine max	XP_003526785.1	532
CYP71A1	Glycine max	XP_003526991.1	519
CYP85A	Glycine max	XP_003544008.2	496
CYP71A1	Glycine max	XP_003549825.1	514
CYP85A	Glycine max	XP_003554965.1	465
CYP89A2	Glycine max	XP_003556183.1	500
CYP89A2	Glycine max	XP_003556196.1	512
CYP714A1	Glycine max	XP_014630399.1	532
CYP734A1	Glycine soja	KHN02133.1	532
CYP89A2	Glycine soja	KHN08060.1	319
CYP85A	Glycine soja	KHN08349.1	464
CYP71A1	Glycine soja	KHN10452.1	437

NADPH-cytochrome p450 reductase	Glycine soja	KHN39959.1	689
CYP89A2	Glycine soja	KHN42995.1	364
CYP716B2	Glycine soja	KHN48077.1	425
CYP716B1	Hevea brasiliensis	XP_021636104.1	492
CYP89A2	Hevea brasiliensis	XP_021673274.1	516
CYP709B2	Hevea brasiliensis	XP_021676114.1	522
CYP98A2	Juglans regia	XP_018843655.1	564
CYP714A1	Lupinus angustifolius	XP_019417080.1	537
CYP85A	Lupinus angustifolius	XP_019442760.1	465
CYP71A1	Lupinus angustifolius	XP_019457306.1	514
CYP71A26	Lupinus angustifolius	XP_019463598.1	505
CYP51	Macleaya cordata	OVA07832.1	461
CYP51	Macleaya cordata	OVA17354.1	488
CYP709B2	Manihot esculenta	XP_021620148.1	523
NADPH-cytochrome p450 reductase	Medicago truncatula	XP_003602898.1	692
CYP98A37	Medicago truncatula	ABC59086.1	509
CYP83B1	Medicago truncatula	XP_003589391.2	515
CYP71B37	Medicago truncatula	XP_003589394.2	514
CYP714A1	Medicago truncatula	XP_003602453.1	524
CYP97B1	Medicago truncatula	XP_003610974.1	574
CYP71A26	Medicago truncatula	XP_013457072.1	522
CYP71A3	Medicago truncatula	XP_013457073.1	521
CYP71A23	Medicago truncatula	XP_013457721.1	495
CYP71A26	Medicago truncatula	XP_013457724.1	500
CYP709B2	Medicago truncatula	XP_013469137.1	518
CYP51	Pisum sativum	BAR45707.1	489
CYP89A2	Populus euphratica	XP_011015670.1	512
CYP89A2	Populus trichocarpa	XP_002306325.2	507
CYP98A2	Populus trichocarpa	XP_002308860.2	508
CYP709B2	Quercus suber	XP_023876027.1	518
CYP71A1	Quercus suber	XP_023886322.1	515
CYP714A1	Theobroma cacao	XP_007011424.1	523
CYP85A	Theobroma cacao	XP_007032850.2	465

CYP709B2	Theobroma cacao	XP_017969771.1	522
CYP71A26	Trifolium pratense	PNX77318.1	439
CYP734A6	Trifolium pratense	PNX93612.1	436
CYP71A26	Trifolium pratense	PNX96961.1	521
CYP71A26	Trifolium pratense	PNY10523.1	434
CYP97B2	Vigna angularis	XP_017407829.1	576
CYP71A1	Vigna angularis	XP_017420230.1	528
NADPH-cytochrome p450 reductase	Vigna angularis	XP_017422031.1	691
NADPH-cytochrome p450 reductase	Vigna radiata	NP_001304239.1	690

Table S5. The expression pattern of differentially spliced isoforms of transcriptional factors involved in seed formation and development.

Gene ID	Expression in（FPKM）	nr_annotation
F01.PB1652	24.23	Zinc finger protein 207
F01.PB2367	7.52	C2H2-type zinc finger protein
F01.PB1740	37.08	RNA-binding family protein with retrovirus zinc
F01.PB1355	2.99	serine/arginine-rich splicing factor RS2Z33 isoform
F01.PB1978	12.25	zinc finger CCCH domain-containing protein 13-like
F01.PB4108	0.53	zinc finger CCCH domain-containing protein 13-like
F01.PB2425	24.87	Zinc finger C-x8-C-x5-C-x3-H type family protein
F01.PB1403	5.03	Zinc finger C-x8-C-x5-C-x3-H type family protein
F01.PB2593	13.82	RING finger and CHY zinc finger domain-containing
F01.PB2608	5.67	RING finger and CHY zinc finger domain-containing
F01.PB3148	22.94	RING finger and CHY zinc finger domain-containing
F01.PB4436	0.20	RING finger and CHY zinc finger domain-containing
F01.PB5109	11.26	zinc finger CCCH domain-containing protein 64-like
F01.PB2932	0.80	zinc finger CCCH domain-containing protein 64-like
F01.PB3615	13.82	squamosa promoter-binding-like protein 12-like
F01.PB5467	0.00	squamosa promoter-binding-like protein 12-like
F01.PB4935	37.32	transcription factor bHLH144-like

F01.PB2402	3.14	transcription factor bHLH144-like

Supplementary Data.

The nucleotide sequences of DXSand DXR in the transcriptome of *C. obtusifolia*

>F01.PB17587(DXR)

ATACACATTTCAGTTTCTGATTCTTTTCCCCTCTTCCAATTGAAGTGGGTTCAGTTCTATTGTTTTCTTGCGAT

GGCTCTGAATTTGCCTTCTCCAGCTGAAGTCAAGTCTGTATTTTTCTCCGATTCCTTCAAGTCTACCAGACAT

GCAAAATTCCCAGGTGGTTTTGCTTTGAAGAGAAAAGATTGTGGAACAACGGTTGGAAGACAAGTTTCCT

GCTCCGTGCAGACACCACCTCCAGCCTGGCCAGGAAGAGCGGTTCCTGAGACCAGTCGCAAGACATGGG

AAGGCCCAAAACCCATCTCTATCGTAGGCTCTACTGGTTCCATTGGAACTCAGACACTGGATATAGTGGCTG

AGAATCCAGATAAGTTCAGAGTTGTGGCACTTGCAGCTGGCTCAAACATTAAACTTCTTGCAGATCAGGTA

AAGACATTCAAGCCTCAACTTGTTGCTGTTAGAAATGAGTCCCTTATTGATGAACTCAAAGAGGCTTTGGCT

GACGTTGAACATAAACCTGAGATCATCCCTGGAGAACAAGGAGTTATTGAGGTTGCTCGTCACCCTGATGC

AGTCACTGTTGTTACGGGAATAGTAGGTTGTGCAGGACTGAAGCCAACAGTTGCTGCTATAGAAGCAGGG

AAAGACATAGCTTTGGCCAACAAAGAGACACTGATTGCTGGAGGTCCTTTTGTCCTTCCTCTTGCTCAAAA

GCATAACATAAAAATTCTTCCTGCTGATTCAGAACATTCTGCCATTTTTCAGTGTATCCAGGGGTTGCCAGAA

GGTGCACTTAGGAGAATTATTTTAACTGCATCTGGAGGTTCTTTCAGGGATTGGCCTGTTGAAAAACTGAA

AGACGTTAAAGTTGCTGATGCATTAAAGCACCCTAACTGGAATATGGGGAAGAAGATAACTGTGGATTCTG

CTACCCTTTTCAACAAGGGCCTGGAAGTAATTGAAGCGCATTACTTGTTTGGTGCTGAATATGATGATATTGA

GATAGTCATTCATCCACAATCTATCATACACTCAATGATTGAAACACAGGATTCATCAGTTTTGGCACAGTTG

GGGTGGCCTGATATGCGCTTGCCAATTCTGTATACAATGTCTTGGCCAGATAGAATTTATTGCTCTGAAGTAA

CTTGGCCTCGTCTTGATCTTTGCAAGCTTGGTTCTTTAACATTTAAAGTTCCGGACAATGTGAAGTATCCATC

GATGAATCTTGCCTATGCTGCTGGCCGTGCTGGAGGCACCATGACTGGAGTTCTCAGTGCAGCAAATGAGA

AAGCCGTAGAAATGTTTATTGATGAAAAGATAAGCTATCTGGATATTTTCAAAGTTGTGGAGCTAACATGCG

AGCAACATCAAAATGAATTAGTAACTTCACCTTCACTTGAAGAGATTATTCATTATGATTTGTGGGCTAGAAA

ATATGCTGCTAGCTTGCAAATCTCTTCCAAAAAGGCTCCTATTCCTGCATGATACTGGAAGGAATTAACAACT

GGGGGATTGATTTCACCTTTTTTCTGTTATCATATTGAGAATGAGTGGATATGTAAAGCATCTACTTCTACAAT

TGTATTTGATCATACCCCAGTTAGAAAAAAATGGTTTTGATTAATGAAAGATTAGATTTTGAT

>F01.PB29801(DXS)

CCCACTCAAAGTTTCAAACTTTCGTCCATTTCTCAATACCCATCTGCTTCATTTCTCTGTTTCTCTTCAATTAGG

TGTGTGTGTGTGTGTGTATCGCATATATACTCTGTTTTCTTTTCTGGGTTTTTCTCCTTTTCAGCTCTGATTGTG

TTTTCCAGAATCTTCTAGAGCTCTCTCTTTGATTCTCTTCAATATGGCTCTTTGCACATTCTCATTTCCTCAAAG

ATCTCTTCTGGGTTCTCACTCTCATTGCGGAGCAGATCTGTACGACTGGCAATGGCAATCTCTACACACCCAC

TCTCAGGTGAAGAAGAGGACGGTTCGAGTATGTGCATCACTATCAGAAAGAGGAGAGTATGCTTCCCGGA

AACCACCAACTCCATTACTGGACACCATAAACTACCCAATTCACATGAAAAATCTCTCTACCAAGGAGCTGA

AACAACTTGCTGATGAACTGCGTTCTGATGTCATTTTCAATGTTTCAAAAACTGGGGGCCATCTGGGTTCAA

GCCTTGGTGTGGTGGAGCTTACGGTTGCTCTCCACTATGTATTCAATGCCCCACAAGATAAGATATTGTGGG

ATGTTGGTCATCAGTCTTACCCACACAAGATACTCACCGGAAGGAGGGATAAGATGCACACCATCAGGCAG

ACAAATGGGTTATCTGGGTTTACCAAACGGTCTGAGAGTGAATTTGATTGTTTTGGAACTGGTCACAGCTC

AACAACTATATCAGCAGGACTAGGTATGGCTGTTGGGAGAGACCTGAAGGGAGGGAAGAATCATGTAGTT

GCTGTTATTGGCGATGGCGCCATGACAGCAGGACAAGCCTATGAAGCCATGAACAACGCTGGATATCTCGA

TTCTGACATGATTGTTATTCTAAATGATAACAAACAGGTTTCTCTTCCAACTGCTAATCTTGATGGTCCTATAC

CACCTGTTGGAGCTTTAAGTAGCGCTCTCAGTAGGCTGCAATCAAACCGACCTCTTAGAGAATTGAGAGAG

GTTGCCAAGGGAGTAACTAAGCAAATTGGTGGCCCTATGCATGAGTTGGCTGCAAAAGTTGATGAGTATGC

TCGTGGTATGATCAGTGGCTCCCGCTCAACGCTATTCGAGGAACTTGGACTCTACTATATTGGTCCTGTTGAT

GGTCATAACATAGATGATCTTGTTGCCATTCTCAATGAAGTTAAGAGTACTAAAACAACTGGTCCTGTATTGA

TCCATGTTGTCACTGAGAAAGGCCGTGGATATCCATATGCCGAAAAAGCAGCAGACAAATACCATGGAGTT

ACTAAGTTTGATCCAGCAACTGGAAAGCAATTCAAAGGCAAGTCTACCACTCAATCTTACACAACATACTTT

GCAGAGGCTTTGATTGCAGAAGCAGAAGCTGACAAAGATATTGTTGCAATCCATGCCGCAATGGGAGGTG

GAACCGGCATGAATCTCTTCCTTCGCCGTTTCCCAACAAGATGCTTTGATGTGGGGATAGCAGAACAACATG

CTGTTACTTTTGCTGCTGGTCTGGCTTGTGAAGGTCTCAAGCCTTTCTGTGCAATTTACTCATCATTCATGCA

AAGGGCTTATGACCAGGTGGTCCATGATGTGGATTTGCAGAAGCTGCCAGTAAGATTTGCAATGGACAGA

GCTGGACTAGTTGGAGCAGATGGTCCAACACATTGTGGTTCTTTTGATGTCACTTTCATGGCATGCCTCCCT

AACATGGTGGTGATGGCTCCTTCTGATGAATCTGAGCTTTTCCATATGGTCGCCACAGCTGCAGCCATTGAT

GATCGCCCCAGTTGCTTCCGGTACCCAAGGGGAAATGGCATTGGTGTTGAACTACCCCCAGGGAACAAAG

GCATTCCTCTAGAGATTGGAAAGGGTAGGATTTTGATTGAAGGGGAAAGAGTGGCCCTCTTGGGCTATGG

ATCTGCTGTTCAGAACTGTTTGGCTGCAGCTTCCTTAGCGCAACGCCACGGCTTGCAAATAACCGTTGCAGA

TGCAAGATTCTGCAAGCCATTGGATCGCGCCCTTATTCGCAGTCTGGCAAAGTCACATGAGGTTTTGATCAC

CGTGGAGGAAGGATCGATCGGAGGATTTGGGTCTCATGTTGCTCAATTCATGGCCCTTGATGGTCTTCTTGA

TGGCAAACTAAAGTGGAGGCCAATAGTTCTTCCCGATCGCTATATCGACCATGGATCGCCTGATGACCAATT

GTCTCTAGCTGGTCTGACACCATCTCACATAGCAGCAACAATATTCAACATTCTTGGACAGACAAGAGAGGC

ATTAGAGGTTTTGTCATAAAAAGGAAAGACTAATGGGGTTCAACATTCCCCACTATGTAAAAAGCATAAATT

AGTAATGTAAAATAGTTCTTTTGTAATGCAGAGAAGTAATATGTTAGTATGTGAGTAATGGAGGTTGTTCATA

GCAAAATGAATTAACCAAAAAAAAAAAAAAAAGGAGTTTTCTATTGAGTTC

Amino acid sequence of CYPs used to phylogenetic analysis from *C. obtusifolia*

>F01.PB16738_Co-CYP450-1

LYNKGNRVAQELDQFFDEVIEQHINRRVKQDLDCLVNDDDDGEEQSDDFVDVLLRIQKR

NITGFPVDITTIKALILDMFAAGTDTTSTVVEWAMTELLRHPRVMKKLQDDMRKVVGSR

THITEEDLVEMHYLKAVLKETLRTHSPVPLLVPRESMQDMKLNGYDIKAGTQVIVNAWAI

AHDASIWDHPEEFKPERFLNNSIDFKGQDFELIPFGAGRRGCPGTAFAMAVNEVVVANLV

HQFDWALPAGKELDMAETAGLTIHRKFPLIAIPSPRKA*

>F01.PB25462_Co-CYP450-2

MATAAISHLSTFPNANSHPKHNRGFSPLSSCVSHFPISIHFRSSMPRGCPVRCQSIDTNERK

SDRNLLDNASNLLTQLLSGGSIGSMPTAEGAVTDLFDRPLFFSLYDWFLEHGSVYKLAFGP

KAFVVVSDPIVARHILRENAFCYDKGVLADILEPIMGKGLIPADLDTWKQRRRVIAPGFHT

SYLEAMIKIFTDCSERTILKFNKLLEGESPNDQKSIELDLEAEFSSLALDIIGLGVFNYDFGS

VTKESPVIKAVYGTLFEAEHRSTFYIPYWKIPLARWIVPRQRKFQSDLKIINSCLDGLIRNA

KESRQETDVEKLQQRDYLNLKDASLLRFLVDMRGADVDDRQLRDDLMTMLIAGHETTA

AVLTWAVFLLAQNPTKMKKAQAEVDSVLGEGRPTFESLKKLQYIRLIVIEALRLYPQPPLL

IRRALKSDVLPGGYKGDKDGYAIPAGTDVFISVYNLHRSPYFWDSPNDFEPERFMVQRKN

EDIEGWAGFDPSRSPGALYPNEVISDFAFLPFGGGPRKCVGDQFALMESTVALALLLQKFD

VQLKGTPQSVELVTGATIHTKNGLWCKLSKRSNLHH*

>F01.PB15276_Co-CYP450-3

EERGKKFRKLLVELGEVIGKFVVGDYIPWLQWLNQISGFYNRGNRVAQELDQFLNEVIEE
HMSTNGEGQSDDFVDVLLRIQKTNITGFPIDTTIIKALILNMFTAGTDTTSTLLEWEMTEL
LRHPRVMKKLQDEMRKVVGSRTHITEEDLVEMHYLKAVLKETLRTHPPVPLLVPRESMQ
DINLNGYDIKAGTHVIVNAWAIAHDASIWDHPEEFKPERFLNSSIDFKGQDFELIPFGAGR
RGCPGTAFAMAVNEMVVANLVHYFDWALPAGKELDMAETAGVIIHRKFPLTAIPSPRKA*

>F01.PB19520_Co-CYP450-4

AHTAFGSSFAQGKEVFIAQRQLQQHCVASASDIFIPGTQYLPTRSNLKTWQLDWKIKKSL
KQIMESRLNSQSSSSYGDDLLGVMLEAAETEKLNSGPKFRMEEIIEECKTFFFAGHETTSN
LLTWTVFLLSIHKEWQERLRQEVLNNCGMEIPDAEVLSKLKMVNMVLLEALRLYCPAIQ
LIRETSEDMKLGNLRIPKHTCVTIPVATIHRKREYWGEDANEFKPERFMNGVSKAASHPN
AMIAFSIGPRACIGQNFAMLEAKTVMVLILQRFSWSLSPHYKHAPANNLTLQPQFGLPVIL
KPLHS*

>F01.PB13542_Co-CYP450-5

MEVVIEFVKLVSVVGVVGILSWLVYGYSSVWVKSERVRRKLRMQGIRGPSPSFLYGNLP
HMHKLQSQAKLTNTTISTTNNHLFLAHDYTSTLFPYFEHWRKQYGQLYTYSTGMKQHLY
VNEAELVREMNQCITLNLGKPSYVTNKLAPLLGNGILRANGLSWAHQRKLVAAEFYMDK
VKGMVGLMIESAQPLLTKWEQLIEAHEGGSSDNGTEVKVDADLRGFSADVISRVCFGHS
YSKGKEVFSKLRSIQKAMSTQGGFQLFGVSGIRGMLHYLWSTKKQNQISSLEKEVESIIW
ELVEERRRKQSEYSSAAAEKDLMQLLLEAAESDQSSDHEGKEFSKRSFIVDNCKNIYFAG
HETTAVAASWCLMLLALHPQWQTSIRNEVAQLCPNGIPDADSLPHLKTVTMVIQETLRLY
PPAAFVSREAYEDVQIGNIKVPKGVCLWTLIPTLHRDPEIWGADANEFKPERFSEGVSKAC
KFPQAYVPFGLGTRLCLGKNFAMVQLKVVLALIISNFSFSLSPSYTHSPAYRMIVEPGHGV
YILIRKI*

>F01.PB15419_Co-CYP450-6

IKFEVSEAEGGRRIHFKKNDNFSGNFETSRNLLLLVITNTCTSNLTHLPFQCHFQMVFQFK
NHQQLTSFSSKIPVIGNLHQLGTCPHRALQSLAQKYGPMMLLHFGKVPVLVVSSADGAC

DVMKTHDLVFSNRPRRRVADILLYASKDIASAQYGEYWRQIRSIGVLHLLSNKRVQSLRG

VREEETKIMMESLEHSASRNLEVNLSEEFSKVTNDIICRVALGRKYSGGERGKKFQKLLV

DFGEVLGTFDVGDYIPWLEWLSNMSGLYNKGNRVAQELDQFFDEVIEQHINRRVKQDLD

CLVNDDDDGEEQSDDFVDVLLRIQKRNITGFPVDITTIKALILDMFAAGTDTTSTVVEWA

MTELLRHPRVMKKLQDDMRKVVGSRTHITEEDLVEMHYLKAVLKETLRTHSPVPLLVPR

ESMQDMKLNGYDIKAGTQVIVNAWAIAHDASIWDHPEEFKPERFLNNSIDFKGQDFELIP

FGAGRRGCPGTAFAMAVNEVVVANLVHQFDWALPAGKELDMAETAGLTIHRKFPLIAIPS

PRKA*

>F01.PB11581_Co-CYP450-7

MGVLMAIIIGVLFLLCFCSALLRWNQLRFSQKGLPPGTMGWPIFGETTEFLKQGPSFMKN

QRARFGSFFKSHILGCPTIVSMDAELNRYILMNESKGLVPGYPQSMLDILGKSNIAAVHGS

THKYMRGALLSIISPSMMKHHLLPKIDHFMRHHLSNWDNQVINIQHKTKQMAFLSSLKQI

AGIESTSPISHNFMSEFFKLVLGTISLPIDLPGANYRRGVQARKNIVSIVRDLVEERRRVSGE

NHQDMLGFLMGKDDDDDGGGRRYKLNDEEIIDLIITIIYSGYETVSTTSMMAVKYLHDHP

KVLQQLRKEHMAIRERKKPEDPIDCEDLKSMKFTRAVIFETSRLATIVNGVLRKTTQDME

LNGYLIPKGWRIYVYTREINYDPFLYPDPLTFNPWRWLDKSIESQSYFLIFGGGTRQCPGK

ELGIAEICTFLHYFVTRYRWEEVGGEKVMKFPRVEAPNGLHIRVSSY*

>F01.PB20786_CO-CYP450-8

ALLVILYSSIILLLLLIIHTRKRRGTSVPPGPPGLPFIGNFHQLHNSAPHHLILCQLSKHYGPL

MSLRLGTRPALVVSSARMASHILKTHDLTFASRPSLLGQHKLSYNGLDLAFSPYTHYWKE

MKKLCVLHLFNPRRIHSFCPIREDEVLRMITKISQSHGLINLSETSMSFTSSLICRIAFGKRY

EEDQGSRFHIGLLTEAQALLAEFYLSDYIPWLGWVDRLTGKLWRLDQIFNKLDMFYQQVI

NDHLDQSNQQQRDIIDIFLQIIHSHNSTPFHLTMDHIKALLMNIFIAGTDTSAATIVWAMTS

LIKNPGVMKKAQDEIRSVYGEKDFIREEDIQRLPYLKAIVKETLRLFPPSPLLVPRESMQK

CNIEGYEIQPKTLVFVNAWAIGRDPETWEEAEQFNPDRFFKCEIDFKGQDFELIPFGAGRR

MCPGMQMGVVTVELALVNLLHSFDWDLPDGIHKHDIDTQIKPGITTHKKIDLCLVAHSHT

KTR*

>F01.PB47552_CO-CYP450-9

TLFMGVTMAIEINAFSAICFLITTLLLVLKLTKRSSKSNVPPSPPKLPLIGNLHQLGSLPHRS
FQTLSRKYGPLMLLHLGQTPTLVVQSSEMAREMTKAHDVVFATRPQTTAAKIFFYQGKD
VSFTRYGEEWRQKRKICVLQLLSPKRVQSFQFIREEEVAELVHNIRQKCGGGGSVINLTQM
TIEATNNIVSRCVLGRKYDAPSGSGSFGDVARKVLSQFSDFSVGDFFPSLGWIDFLTGLISK
LKGTLGELDAVFDDVIAQHKKKSNIIEDEERKDFVDILLEIQKNGMLDFELTNDHIKGVL
MDLFLGGSDTSSATIEWAMAELMRSPTEMRKVQDEVRRVVGHKSKVEEADVKQMKYL
GFVVKEALRLHPPNLIIPREARSSVKLGGYDIPSKATVFINVWAIQRDPQVWERAEEFIPER
FENSEIDFKGQDFEFIPFGSGRKGCPGMTFGVTMVEYVLANVLYWFDWKLPPTAQDVDM
NEGYGLTVTKKEPLLLQPSIFSFSP*

>F01.PB20158_CO-CYP450-10

MALFLIPIAILALLLCFNLYHRLRFRLPPGPRPWPIVGNLYDIKPVRFRCFAEWAQSYGPIIS
VWFGSTLNVIVSNSELAREVLKEHDQQLADRHRSRSAAKFSRDGKDLIWADYGPHYVK
VRKVCTLELFSPKRLEALRPIREDEVTAMVESVFRDSTNPENEGKSILVKKYLGAVAFNNI
TRLAFGKRFVNEEGVMDEQGVEFKAIVSNGLKLGASLAMAEHIPWLRWMFPLEEEAFA
KHGARRDRLTRAIMEEHTQARNLSGGAKQHFVDALLTLQDKYDLSEDTIIGLLWDMITA
GMDTTAISTEWAMAELIKNPRVQQKAQEEMDRVIGFERVMTETDFSSLPYLQCVAKEALR
LHPPTPLMLPHRANANVKVGGYDIPKGSNVHVNVWAIARDPAVWKDPLEFRPERFLEED
VDMKGHDFRLLPFGAGRRVCPGAQLGINLVTSMLGHLLHHFCWAPPEGMKPEEIDMSEN
PGLVTYMRTPLQAVATPRLPSQLYKRVPADM*

>F01.PB15287_CO-CYP450-11

EEAFPALLALLFLLLSLHFITKIIVKTTRKHQNLPPGSLGWPIVGETLEFMRCGGEGNHERF
IRERMEKYDWRVFKTSMLGEAMIVFCGPAGNKLLFSNENKNVQVWWPSSVRKLLRSSL
VTKVGSEAKTMKRLLTTFLNPEALKNYLSKMDAIAHTHIHTHWQGKEQVLVYPTVKKY
TFELACSLFASIEDSIHISKLSSQFDEFLKGVISFPVNFPGSRFHCAMRAANAIRQELKLIIKK
RRVDLEDKSASPTQDLLSHLLLTSDTTGRFLTEMEIIDNILLLLFAGHDTSRSVISSIFKYLG
QFPQVYQQVCKNN*

>F01.PB2613_Co-CYP450-12

RKEQDDVLIPLIRARKKAREERLSKSEEDRCKVVENDVVVPYVDTLFDLELPEEKRKLEE
SELVSVTSEFINAGTDTTATALQWIMANLVKYPEIQERVFEEIKEVMGNKIGEDNEEVKEE
DLNKLGYLKAVILEGLRRHPPGHFLLPHAVSEDVVLNDYLVPKNGTINVMVAEMGWDPK
VWENPMEFKPERFLRSEGEEAFDITGSKEIKMMPFGAGRRICPGYNLAMLHLEYFVANLV
WNFEWKAGNGDVDLSEKQEFTVVMANPLQVQLTRRR*

>F01.PB9468_Co-CYP450-13

MTTFLQTSTMHDSTFWSLQTVLVLLILPIFLFKWYSNSKTTNNLPPSPPKFPVIGNLHQLG
TYPHRTLHSLAQKYGPLMLLHFGKVPVLVVSSADAARDVTKTHDLVFSNRPRRRVADILT
YGSIDIAFSGYGEYWRQIRSIGVLHLLSNKKVQSIRGVREEETKILMENINHSASTNLQVN
LSEEFFRLANDIICRVALGNKYSGEERGKKFRKLLVELGEVIGKFVVGDYIPWLQWLNQIS
GFYNRGNRVAQELDQFLNEVIEEHMSTNGEGQSDDFVDVLLRIQKTNITGFPIDTTIIKALI
LNMFTAGTDTTSTLLEWEMTELLRHPRVMKKLQDEMRKVVGSRTHITEEDLVEMHYLK
AVLKETLRTHPPVPLLVPRESMQDINLNGYDIKAGTHVIVNAWAIAHDASIWDHPEEFKPE
RFLNSSIDFKGQDFELIPFGAGRRGCPGTAFAMAVNEMVVANLVHYFDWALPAGKELDM
AETAGVIIHRKFPLTAIPSPRKA*

>F01.PB11533_Co-CYP450-14

GEEWRQKRKICVLELLSLKRVRSFQFIREEEASDLVTKIRESCENNIINGSPALINLTPMLIA
ATSNVVSRCVLGQKSEKADGKSSFGDVARKMMIQLAAFSFGNFFPSLGWMDAVSGLIPK
LKATSVELDTFFDEIIAQHKMAKTNDDESEKKDFVDILLQLQDDDMLEFKLTHQDLKGMI
ADMFVGGGDTTSTALEWTFAELLKNPIAMKKVQEEVRRVVGHKSKIDENDVRQMKYLE
CVVKESLRLHPPLPLLVPRETRSIAKLGEYDIPPQTSVFLNAWAIQRDPELWEKPEEFIPERF
ENSEVDFKGQDFQLVPFGFGRRGCPGMSFGVASVEYFLAVLLFWFDWKLPSGTTTTSSQD
IDMSEIYGLTVNKKVPILVEPIPYSFGPQL*

>F01.PB22595_Co-Monooxygenase-1

LTASRCLLGREVRDKLFDDVSALFHDLDNGMLPISVLFPYLPIPAHRRRDQARKKLSEIFA
SIITSRKSTGKSENDMLQCFIDSKYKDGRPTTESEVTGLLIAALFAGQHTSSITSTWTGAYL
LCNKQYLSAVMEEQKELMGKHGDRVDHDILAEMDVLYRCIKEALRLHPPLIMLLRSSHS

DFSVKTREGIEYDIPKGHIVATSLLLRTAYLIFSRILIVMILIGLP*

>F01.PB4860_Co-Monooxygenase-2

MFCLLVFMCFGERLDEKQIREIEFVQRRLLLNFNKYSMLNIMPRITRILFRKLWEELLQVR

KEQDDVLIPLIRARKKAREERLSKSEEDRCKVVENDVVVPYVDTLFDLELPEEKRKLEES

ELVSVTSEFINAGTDTTATALQWIMANLVKYPEIQERVFEEIKEVMGNKIGEDNEEVKEED

LNKLGYLKAVILEGLRRHPPGHFLLPHAVSEDVVLNDYLVPKNGTINVMVAEMGWDPKV

WENPMEFKPERFLRSEGEEAFDITGSKEIKMMPFGAGRRICPGYNLAMLHLEYFVANLV

WNFEWKAGNGDVDLSEKQEFTVVMANPLQVQLTRRR*

>F01.PB24506 _Co-reductase -1

MASSSDLVRALESMLGISMGDSVSDSLLVIATTSVAVVIGLLVFMWKKSSDRSKEVKPLVV

PKSLSVKDEEDDVDVGSGKTRVTVFFGTQTGTAEGFAKALAEEIKARYEKAVVKVVDMD

DYAADDDEYEEKLKKETLAFFMLATYGDGEPTDNAARFYKWFTEGKEEGASWLQQLTY

GVFGLGNRQYEHFNKIGKVIDQKLSDQGAKRLVPVGLGDDDQSIEDDFSAWKESLWPEL

DQLLRDEDDMNTVSTPYTAAIAEYRLVIHDPTITSCYDRNLSMTNGNALFDIHHPCRVDV

AVQRELHKPESDRSCIHLEFDISGTGITYETGDHVGVYAENCDETVEEAGKLLGQNLDLL

FSLHTDNEDGASLGGSLPPPFPGPCTLRAALARYADLLNPPRKAALVALAAHASEPSESER

LKFLTSPQGKDEYSKWVVGSQRSLLEVMAEFPSAKPPLGVFFAAVAPRLQPRYYSISSSPK

FAPQRVHVTCALVYGPTPTGRIHKGVCSTWMKNAVPSEKSRDCSWAPIFIRPSNFKLPADH

LTPIIMVGPGTGLAPFRGFLQERLALKEEGVELGPALLFFGCRNRQMDFIYEDELTNFVEQ

SALSELVVAFSREGPEKEYVQHKMMDKSEYLWSLISQGGYLYVCGDAKGMARDVHRTL

HTIVQQQENVDSSKAEAIVKKLQMDGRYLRDVW*
